# An asymptotic slender-body model for predicting print resolution in extrusion-based bioprinting

**DOI:** 10.1007/s10665-026-10544-0

**Published:** 2026-08-12

**Authors:** Amy V. Tansell, Galane J. Luo, Paraskevi Diakourti, Nasim Mahmoodi, Lauren E. J. Thomas-Seale, Rosemary J. Dyson

**Affiliations:** 1 School of Mathematics, University of Birmingham, Birmingham, B15 2TT UK; 2 Centre for Doctoral Training in Topological Design, University of Birmingham, Birmingham, B15 2TT UK; 3 Department of Mechanical Engineering, School of Engineering, University of Birmingham, Birmingham, B15 2TT UK; 4 Department of Electronic Electrical and Systems Engineering, School of Engineering, University of Birmingham, Birmingham, B15 2TT UK

**Keywords:** Asymptotic analysis, Extrusion-based bioprinting, Non-Newtonian fluids, Printing resolution, Rheology, Slender-body theory

## Abstract

Bioprinting enables fabrication of three-dimensional, patient-specific, tissue-like structures, for which printing resolution and printability are key determinants of construct fidelity. Achieving consistent control of printed filament dimensions remains challenging due to its strong dependence on interacting process parameters, while existing print-and-test approaches are often time-consuming, expensive, and specific to particular materials and experimental set-ups. This study proposes a generalised, physics-based model to predict printed filament diameters for moderately shear-thinning materials under pneumatic extrusion-based bioprinting. By adopting an arc length coordinate system and exploiting the slender geometry of the extruded filament, asymptotic techniques are employed to reduce the incompressible Navier–Stokes equations to a closed system describing filament radius and centre-line orientation. Non-Newtonian behaviour characteristic of bioinks is captured by modelling the material as a power-law fluid. Applied prior to printing, the model enables identification of process parameter combinations corresponding to a bioink’s window of printability, reducing reliance on print-and-test methodologies. Preliminary validation using pneumatically extruded Nivea Crème demonstrates good predictive capability. This work highlights the value of theoretical modelling for supporting optimisation-driven design in extrusion-based bioprinting.

**Supplementary Information:**

The online version contains supplementary material available at 10.1007/s10665-026-10544-0.

## Introduction

Pneumatic extrusion-based bioprinting applies pressurised air to drive bioinks through a nozzle, generating continuous filaments. It is a popular choice of bioprinting method on account of its compatibility with a broad range of viscosities and the high cell densities achieved within printed constructs [1, 2]. Hydrogels and their precursors are commonly employed as bioinks, as their physicochemical properties support printability while providing an extracellular-matrix-like microenvironment beneficial to cell viability [3, 4]. Consequently, bioprinting enables the fabrication of three-dimensional (3D), patient-specific, tissue-like structures, emphasising its potential for tissue replacement and the need for systematic optimisation of its design strategy [5].

To evaluate printing performance within this framework, we first define key metrics used throughout this work. *Printability* is defined here as the extent to which a printed construct deviates from its intended 3D model data for a given application. This is characterised through three components: extrudability, shape fidelity, and structural integrity [6]. Extrudability refers to the ability of a bioink to be continuously and controllably extruded through a nozzle; shape fidelity describes how closely the printed filament geometry (e.g. cross-sectional area) matches that specified in the 3D model; and structural integrity denotes the ability of the printed construct to maintain its intended structure after deposition under its own weight.

*Printing resolution* is defined as the smallest achievable printed filament diameter [7, 8]. In this work, we further define the *optimal resolution* to be a printed filament diameter that closely matches the nozzle tip diameter.

These metrics are inherently linked: resolution is constrained by printability, as nozzle geometry (and thus achievable filament diameter) is limited by bioink extrudability, while sufficient structural integrity is required to preserve filament dimensions post-deposition. As such, printing resolution is of critical importance in the fabrication of tissue scaffolds and constructs intended to replicate the mechanical properties of native tissues. Owing to its sensitivity to the experimental set-up, resolution can be a complex parameter to control, with the greatest influence arising from controllable process parameters, including applied pressure, nozzle travel speed, and nozzle geometry [9]. Therefore, achieving a suitable trade-off between printability and resolution is essential. By identifying suitable combinations of process parameters that yield filament diameters within a small tolerance of this optimum, we isolate the limiting operational cases that define the printable design space, referred to as the ‘*window of printability*’ of a bioink [10, 11].

In extrusion-based bioprinting, these combinations of process parameters associated with optimal resolution are typically established via *print-and-test* (*characterisation*) methodologies. While effective, these approaches are often time-consuming, expensive, and specific to particular materials and experimental set-ups. As a result, empirical modelling has emerged as a promising alternative, seeking to relate process and material parameters to filament shape fidelity and resolution [11–13]. However, being fundamentally rooted in experimental observation, their predictive capability and generalisability are inherently limited [9, 10]. In particular, existing approaches do not explicitly capture the extensional fluid flow governing filament formation during pneumatic extrusion.

To reduce reliance on empirical estimation, we propose a physics-based approach that exploits the governing fluid mechanics of pneumatic extrusion to derive generalised time-dependent and steady-state mathematical models capable of predicting printed filament resolution for moderately shear-thinning materials, here defined as fluids with a flow behaviour index $0.40\le n \le 0.65$. Unlike existing empirical approaches, the proposed framework explicitly captures the extensional flow of the extruded filament and enables theoretical prediction of print resolution from governing physical principles.

While the ultimate goal of extrusion-based bioprinting is the controlled deposition of biologically relevant materials, key aspects of filament formation during extrusion and deposition are governed by fluid mechanical behaviour. To isolate these mechanisms, the present work focuses on model fluids with well-characterised and reproducible rheological properties. As such, the term ‘bioprinting’ therefore refers to the extrusion-based manufacturing process itself rather than a specific material, providing a foundation for understanding and predicting printability and resolution for more complex bioinks.

By adopting an arc length coordinate system and exploiting the slender geometry of the extruded filament, asymptotic techniques are employed to reduce the incompressible Navier–Stokes equations to a closed system describing the filament radius and centre-line orientation [14, 15].

In doing so, we apply the classical extensional flow framework originally developed by Trouton [16], and adapt well-established extensions for curved centre-line geometries to the context of extrusion-based bioprinting and to materials that exhibit non-Newtonian behaviour [14, 17]. To capture the shear-thinning, non-Newtonian behaviour characteristic of bioinks used for extrusion-based bioprinting, we model the filament as a *power-law fluid* [10]. While complex fluid behaviour—such as yield stress, viscoelasticity, and post-extrusion cross-linking—may influence filament morphology, this paper focuses on the dominant fluid behaviour governing filament printability and resolution under steady pneumatic extrusion [18].

When applied prior to printing, the model is capable of theoretically identifying combinations of process parameters corresponding to optimal resolutions and thereby establishing a bioink’s window of printability [10]. In contrast to experimentally derived windows of printability reported by Paxton et al. [10], the proposed framework provides a generalised, physics-based methodology for identifying printable operating regimes across different materials and process conditions.

In previous work, flow within the nozzle was modelled to determine the extrusion speed at the nozzle tip, producing an empirical relationship that links extrusion speed to the applied air pressure [19]. In this paper, that empirical expression is used as an input to the new theoretical model, which goes beyond the nozzle and captures the behaviour of the extruded filament after it leaves the nozzle. This enables the model to track the evolving geometry and flow dynamics of the filament from the nozzle tip to the build platform, ultimately allowing the print resolution to be predicted. By introducing this additional theoretical component, we move beyond the simplified flux-based assumption used previously—where material is assumed to be deposited directly onto the build platform without further evolution—and instead account for the deformation of the filament during extrusion and deposition. Consequently, the model captures physical mechanisms that were absent from the previous framework and provides a more realistic basis for predicting print resolution and identifying windows of printability.

For preliminary validation, Nivea Crème was pneumatically extruded over a range of applied pressures. Its constant quality and stable composition at high and low temperatures have led bioprinting pioneer REGENHU (Switzerland) to establish Nivea Crème (Beiersdorf Global AG, Germany) as a standard demonstration ink [10]. Importantly, rheological characterisation conducted by Paxton et al. [10] demonstrates that Nivea Crème exhibits shear-thinning behaviour that can be well described by a power-law model, enabling direct extraction of the flow behaviour index *n* used in this work. Experimental measurements obtained under these conditions exhibited good agreement with the model predictions.

Overall, this unique methodology seeks to enhance the comparability and transferability of results across materials and experimental set-ups. By alleviating reliance on print-and-test and highlighting theoretical significance, this framework has potential to improve the efficiency of extrusion-based bioprinting and to support greater design creativity through a user-friendly and sustainable tool for optimising printability and resolution.

The remainder of this paper develops the mathematical model (Sect. 2.1), outlines its numerical implementation (Sect. 2.2), and assesses its predictive capability through preliminary comparison with experimental data (Sect. 3).

## Materials and methods

### Mathematical model

**Fig. 1:**
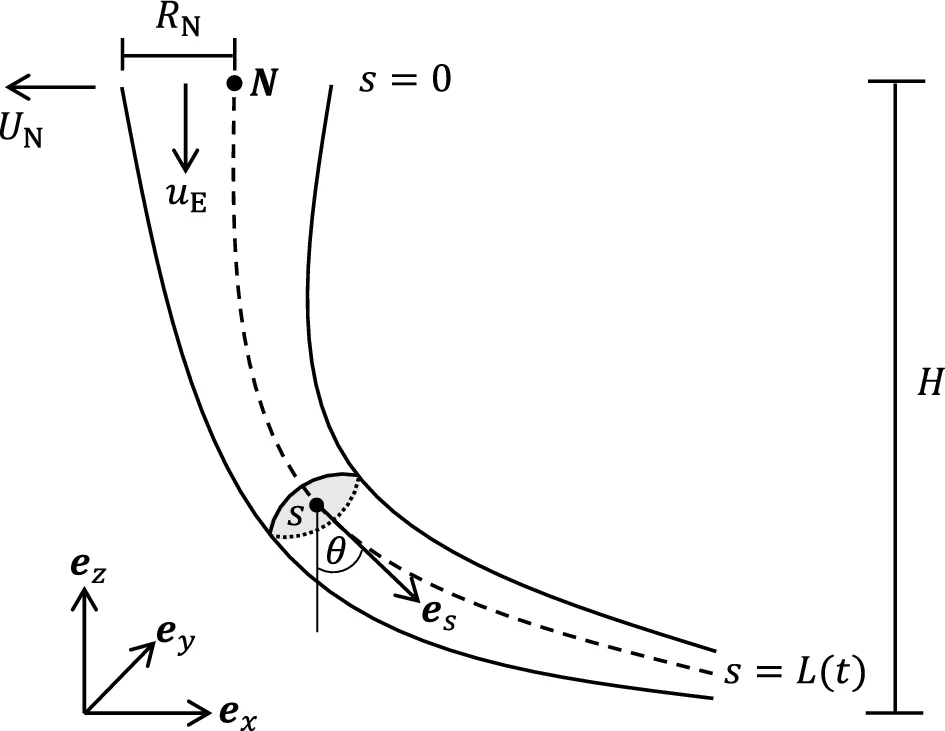
Model set-up, with $R_{\text {N}}$, $U_{\text {N}}$, $u_{\text {E}}$, and *H* the radius, travel speed, extrusion speed, and offset of the nozzle, respectively. Filament centre-line lies in *xz*-plane and material meets build platform at s=L(t), with *s* the centre-line arc length. Centre of nozzle tip position denoted ${\boldsymbol{N}}(t)$ (at s=0). At a chosen *s*, ${\boldsymbol{e}}_{s}(s,t)$ denotes the centre-line tangent vector, with orientation, $\theta (s,t)\ge 0$, from downward vertical [where θ(s,t)=0]

We consider the pneumatic extrusion of a slender filament of hydrogel via a nozzle, with extrusion speed $u_{\text {E}}$, onto a stationary build platform from a height *H*, as shown in Fig. 1. The nozzle moves horizontally with speed $U_{\text {N}}$.

Since the length of the extruded filament is typically much greater than its thickness, we assume a *slender geometry* characterised by a small aspect ratio $\varepsilon ^{n}= R_{\text {N}}/L$, where *n* is the flow behaviour index, introduced here to account for shear-dependent viscosity, $R_{\text {N}}$ the radius of the nozzle tip, and *L* a typical length of the filament. Taking advantage of this slenderness, we model the filament via its curved centre-line, parametrised by an arc length *s*, measured from the nozzle tip to a chosen point, where ${\boldsymbol{N}}(t) = (N_{x}(t), 0, H)$ denotes the position of the centre of the nozzle tip (at s=0). The point of contact with the build platform is located at s=L(t).

We take η to be the apparent viscosity and ρ to be the density of the fluid which is contained within a boundary denoted by r=R(ϕ,s,t), with ϕ the azimuthal angle within the filament cross section, measured anti-clockwise from the centre of curvature. Given that pneumatic extrusion-based bioprinting is characterised by short timescales and high strain rates, and that bioinks typically consist of high water content hydrogels, we reasonably model the bioink as an incompressible fluid [4, 10]. Assuming an incompressible power-law fluid, ρ remains constant and η is described by the constitutive relation

1 $$\begin{aligned} \eta (\dot{\gamma }) = K\dot{\gamma }^{n-1}, \end{aligned}$$

where *K* denotes the consistency index, $\dot{\gamma }$ the shear rate, and *n* is the flow behaviour index.

During extrusion, the hydrogel filament may exhibit die-swell, a phenomenon in which the emerging filament expands to a radius larger than that of the nozzle tip. Although die-swell is often observed when extruding with hydrogels, its underlying mechanics remain complex, and relatively little quantitative research has been conducted to characterise this behaviour [9]. Despite its potential impact on resolution and shape fidelity, the transition region between fully developed pipe flow inside the nozzle and extensional free-surface flow outside is confined to a near-nozzle boundary layer whose characteristic length scale is comparable to the filament radius, i.e. $\delta = {\mathcal {O}}(R_{\text {N}})$ [20, 21]. Since $\varepsilon ^{n}=R_{\text {N}}/L$, the boundary layer length scale relative to the filament length satisfies $\delta /L = {\mathcal {O}}(\varepsilon ^{n})\ll 1$. Consequently, in the asymptotic limit of a slender filament, the influence of this near-nozzle boundary layer on the outer filament dynamics is expected to be negligible.

Assuming the filament maintains an approximately circular cross section, the volumetric flow rate, *Q*, leaving the nozzle remains constant, taking the value $Q=\pi R_{\text {N}}^{2}\bar{u}_{\text {E}}$, with $R_{\text {N}}$ the radius of the nozzle tip and

2 $$\begin{aligned} \bar{u}_{\text {E}} = \frac{1}{A}\iint _{D} u_{\text {E}} \, \text {d}A, \end{aligned}$$

the average extrusion speed across the cross section of the nozzle tip, *D*, with *A* its cross-sectional area. We note that the geometric and mechanical properties of the filament are obtained by taking averages over its cross section.

#### Centre-line geometry and kinematics

We denote the position of the centre-line by ${\boldsymbol{r}}_{\text {c}}(s,t)=(N_{x}(t) + X(s,t),0, H + Z(s,t))$, with respect to fixed Cartesian axes (*x*, *y*, *z*), and assume it lies in the xz-plane (where y=0). To capture the orientation of the filament centre-line, we take $\theta (s,t)\ge 0$ to be the angle between the tangent at *s* and the downward vertical (where θ=0). We assume the property that every *s* along the centre-line is exactly the *centroid* of the perpendicular cross section at that point. As such, we define basis vectors $\{{\boldsymbol{d}}_{1}(s,t),{\boldsymbol{d}}_{2}(s,t),{\boldsymbol{e}}_{s}(s,t)\}$ that move with the filament centre-line, and choose ${\boldsymbol{e}}_{s}$ to point in the tangential direction to the centre-line such that ${\boldsymbol{e}}_{s}\cdot {\boldsymbol{e}}_{z} = -\cos {\theta }$. Consequently, ${\boldsymbol{e}}_{s}$ is given by

3 $$\begin{aligned} {\boldsymbol{e}}_{s}(s,t) = \frac{\partial {\boldsymbol{r}}_{\text {c}}}{\partial s} = \sin {\theta }\,{\boldsymbol{e}}_{x} - \cos {\theta }\,{\boldsymbol{e}}_{z}. \end{aligned}$$

**Fig. 2:**
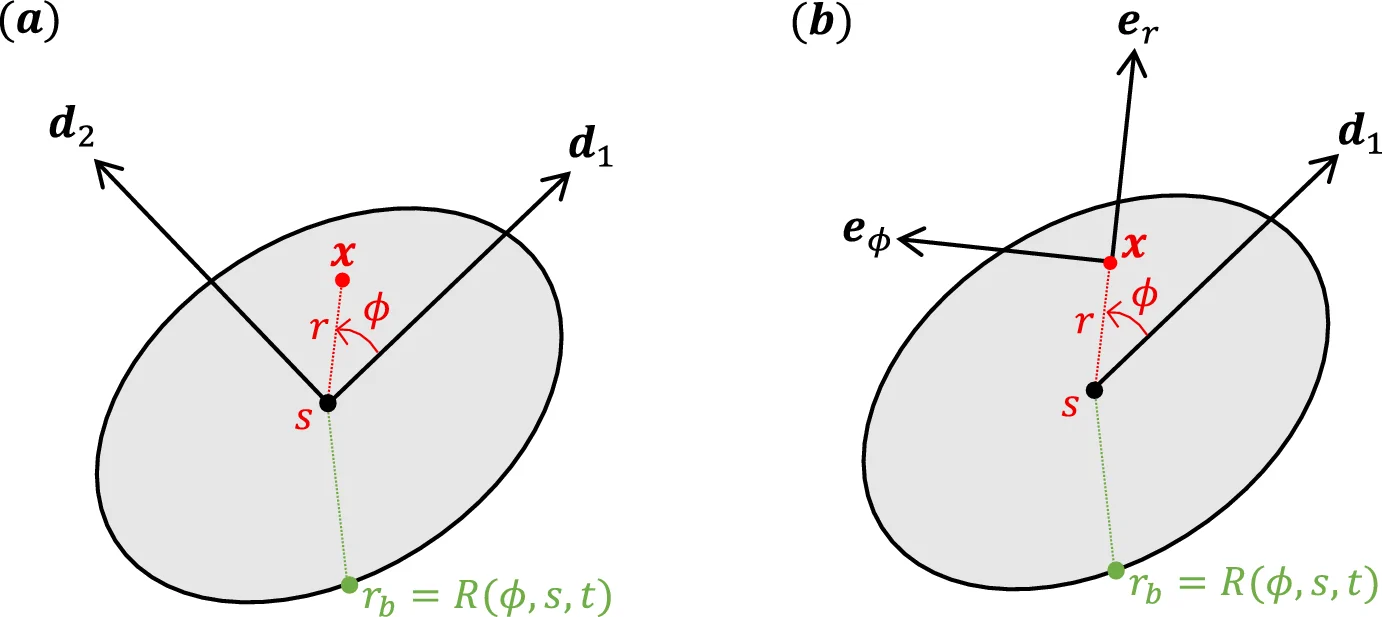
At a chosen *s*, we define cross section (with radius *R*) basis vectors, given by **a** ${\boldsymbol{d}}_{1}(s,t)$ and ${\boldsymbol{d}}_{2}(s,t)$ fixed at centre-line, with ${\boldsymbol{d}}_{1}$ pointing towards centre of curvature and ${\boldsymbol{d}}_{2} = -{\boldsymbol{e}}_{y}$, and **b** ${\boldsymbol{e}}_{r}(\phi ,s,t)$ and ${\boldsymbol{e}}_{\phi }(\phi ,s,t)$, with ${\boldsymbol{e}}_{r}$ pointing in normal direction and ${\boldsymbol{e}}_{\phi }$ in azimuthal direction. Arbitrary point, ${\boldsymbol{x}}$, determined by *r*, ϕ, *s*, where ϕ increases anti-clockwise from ${\boldsymbol{d}}_{1}$ as we look up at the cross section

Lying in the cross section at *s*, we have ${\boldsymbol{d}}_{1}$ and ${\boldsymbol{d}}_{2}$ denoted by

4 $$\begin{aligned} {\boldsymbol{d}}_{1}(s,t) = \cos {\theta }\,{\boldsymbol{e}}_{x} + \sin {\theta }\,{\boldsymbol{e}}_{z}, \quad {\boldsymbol{d}}_{2}(s,t) = -{\boldsymbol{e}}_{y}, \end{aligned}$$

with ${\boldsymbol{d}}_{1}$ pointing towards the centre of curvature, and ${\boldsymbol{d}}_{2} = {\boldsymbol{e}}_{s}\times {\boldsymbol{d}}_{1}$. An arbitrary point in the cross section is then described as a linear combination of these vectors, ${\boldsymbol{x}}=m\,{\boldsymbol{d}}_{1}+n\,{\boldsymbol{d}}_{2}$, where

5 $$\begin{aligned} m = {\boldsymbol{x}}\cdot {\boldsymbol{d}}_{1} = r\cos {\phi }, \quad n = {\boldsymbol{x}}\cdot {\boldsymbol{d}}_{2} = r\sin {\phi }, \end{aligned}$$

as seen in Fig. 2a. The centroid of the cross section is located via the following:

6a $$\begin{aligned}&\begin{aligned} \bar{m} = \int _{A} m\;\text {d}A = 0, \end{aligned} \end{aligned}$$

6b $$\begin{aligned}&\begin{aligned} \bar{n} = \int _{A} n\;\text {d}A = 0, \end{aligned} \end{aligned}$$

that is

7a $$\begin{aligned}&\begin{aligned}&\int _{0}^{2\pi }R^{3}(\phi ,s,t)\cos {\phi }\;\text {d}r\,\text {d}\phi = 0, \end{aligned} \end{aligned}$$

7b $$\begin{aligned}&\begin{aligned}&\int _{0}^{2\pi }R^{3}(\phi ,s,t)\sin {\phi }\;\text {d}r\,\text {d}\phi = 0. \end{aligned} \end{aligned}$$

An arbitrary point in the cross section can also be described via basis vectors ${\boldsymbol{e}}_{r}(\phi ,s,t)$ and ${\boldsymbol{e}}_{\phi }(\phi ,s,t)$, with ${\boldsymbol{e}}_{r}$ pointing in the normal direction, the direction of increasing *r* (with an origin at the centroid), and ${\boldsymbol{e}}_{\phi }={\boldsymbol{e}}_{s}\times {\boldsymbol{e}}_{r}$ pointing in the azimuthal direction, the direction of increasing ϕ, as seen in Fig. 2b. We assume the filament to be approximately axisymmetric about its centre-line. Variation of the triad of basis vectors along the filament is captured via the curvature of its centre-line, κ(s,t). Assuming a centre-line in the xz-plane, κ(s,t) can be expressed as the rate of change of θ(s,t) with respect to the arc length *s*, i.e. $\kappa (s,t)= \partial \theta / \partial s$. From Fig. 1, the geometry of the centre-line is described as follows:

8a $$\begin{aligned}&\begin{aligned} \frac{\partial X}{\partial s}=\sin {\theta (s,t)}, \end{aligned} \end{aligned}$$

8b $$\begin{aligned}&\begin{aligned} \frac{\partial Z}{\partial s}=-\cos {\theta (s,t)}, \end{aligned} \end{aligned}$$

8c $$\begin{aligned}&\begin{aligned} \frac{\partial \theta }{\partial s}=\kappa (s,t), \end{aligned} \end{aligned}$$

and the position of the centre-line rewritten as

9 $$\begin{aligned} {\boldsymbol{r}}_{\text {c}}(s,t) = \left( N_{x}(t) + \int _{0}^{s} \sin {\theta (s',t)}\,\text {d}s'\right) {\boldsymbol{e}}_{x} + \left( H + \int _{0}^{s} -\cos {\theta (s',t)}\,\text {d}s'\right) {\boldsymbol{e}}_{z}, \end{aligned}$$

where the nozzle position in *y*, $N_{y}(t) = 0$. Measured along the centre-line, from the nozzle tip to the point of contact with the build platform, the filament length, *L*(*t*), is then determined via the following condition:

10 $$\begin{aligned} \int _0^{L(t)} \cos {\theta }(s, t)\,\text {d}s = H - R(\phi ,L(t),t), \end{aligned}$$

with R(ϕ,L(t),t) the filament radius on the build platform [at s=L(t)]. We note that we obtain the tangential basis vector, ${\boldsymbol{e}}_{s}$, when we differentiate ${\boldsymbol{r}}_{\text {c}}(s,t)$ with respect to *s*, as required. Here, an arbitrary point in Cartesian space is defined by

11 $$\begin{aligned} \begin{aligned}&x = N_{x}(t) + \int _{0}^{s} \sin {\theta (s',t)}\,\text {d}s' + r\cos {\theta }\cos {\phi }, \\&y = - r\sin {\phi }, \\&z = H + \int _{0}^{s} -\cos {\theta (s',t)}\,\text {d}s' + r\sin {\theta }\cos {\phi }, \end{aligned} \end{aligned}$$

with *r* the radial coordinate in the ${\boldsymbol{e}}_{r}$ direction and ϕ the azimuthal angle in the ${\boldsymbol{e}}_{\phi }$ direction (lying in the cross section at *s*). To ensure a right-handed triad, (r,ϕ,s), ϕ must increase anti-clockwise from ${\boldsymbol{d}}_{1}$ as we look up at the cross section. Equation (11) relates Cartesian coordinates, *x*, *y*, *z*, to the body-fitted coordinates, *r*, ϕ, *s*. This yields the following scaling factors:

12 $$\begin{aligned} L_{r}=1, \quad L_{\phi }(r)=r, \quad \text {and} \quad L_{s}(r,\phi ,s)=1-r\kappa \cos {\phi }. \end{aligned}$$

The associated unit basis vectors in the radial and azimuthal directions are written

13 $$\begin{aligned} \begin{aligned}&{\boldsymbol{e}}_{r}(\phi ,s,t) = \cos {\theta }\cos {\phi }\,{\boldsymbol{e}}_{x} - \sin {\phi }\,{\boldsymbol{e}}_{y} +\sin {\theta }\cos {\phi }\,{\boldsymbol{e}}_{z}, \\&{\boldsymbol{e}}_{\phi }(\phi ,s,t) = -\cos {\theta }\sin {\phi }\,{\boldsymbol{e}}_{x} - \cos {\phi }\,{\boldsymbol{e}}_{y} - \sin {\theta }\sin {\phi }\,{\boldsymbol{e}}_{z}, \end{aligned} \end{aligned}$$

with inverse relationships

14 $$\begin{aligned} \begin{aligned}&{\boldsymbol{e}}_{x}(\phi ,s,t) = \cos {\theta }\cos {\phi }\,{\boldsymbol{e}}_{r} - \cos {\theta }\sin {\phi }\,{\boldsymbol{e}}_{\phi } + \sin {\theta }\,{\boldsymbol{e}}_{s}, \\&{\boldsymbol{e}}_{y}(\phi ,s,t) = -\sin {\phi }\,{\boldsymbol{e}}_{r} - \cos {\phi }\,{\boldsymbol{e}}_{\phi }, \\&{\boldsymbol{e}}_{z}(\phi ,s,t) = \sin {\theta }\cos {\phi }\,{\boldsymbol{e}}_{r} - \sin {\theta }\sin {\phi }\,{\boldsymbol{e}}_{\phi } - \cos {\theta }\,{\boldsymbol{e}}_{s}. \end{aligned} \end{aligned}$$

We express the rate of change of position of the centre-line in a moving basis as follows:

15 $$\begin{aligned} \frac{\partial {\boldsymbol{r}}_{\text {c}}}{\partial t} = v_{r}(\phi , s,t)\,{\boldsymbol{e}}_{r} + v_{\phi }(\phi , s,t)\,{\boldsymbol{e}}_{\phi } + v_{s}(s,t)\,{\boldsymbol{e}}_{s}, \end{aligned}$$

where ${\boldsymbol{v}} = {\boldsymbol{U}} - {\boldsymbol{u}}$, with ${\boldsymbol{U}}$ and ${\boldsymbol{u}}$ denoting fluid velocity relative to some fixed lab frame and the (moving) centre-line, respectively, and has components

16a $$\begin{aligned}&\begin{aligned} v_{r}(\phi , s,t) = \cos {\phi }\left( \cos {\theta }\left( \int _{0}^{s} \frac{\partial \theta }{\partial t}\cos {\theta } \,\text {d}s'- U_{\text {N}} \right) + \sin {\theta }\int _{0}^{s} \frac{\partial \theta }{\partial t}\sin {\theta } \,\text {d}s'\right) , \end{aligned} \end{aligned}$$

16b $$\begin{aligned}&\begin{aligned} v_{\phi }(\phi ,s,t) = -\sin {\phi }\left( \cos {\theta }\left( \int _{0}^{s} \frac{\partial \theta }{\partial t}\cos {\theta } \,\text {d}s'- U_{\text {N}} \right) + \sin {\theta }\int _{0}^{s} \frac{\partial \theta }{\partial t}\sin {\theta } \,\text {d}s'\right) , \end{aligned} \end{aligned}$$

16c $$\begin{aligned}&\begin{aligned} v_{s}(s,t) = \sin {\theta }\left( \int _{0}^{s} \frac{\partial \theta }{\partial t}\cos {\theta } \,\text {d}s'- U_{\text {N}} \right) - \cos {\theta }\int _{0}^{s} \frac{\partial \theta }{\partial t}\sin {\theta } \,\text {d}s', \end{aligned} \end{aligned}$$

using $\text {d} N_{x}/\text {d}t = - U_{\text {N}}$. We note that, in the absence of swirl, the centre-line velocity is independent of ϕ, with any apparent ϕ-dependence arising solely from expressing the velocity in a moving basis. However, if swirl is present, fluid particle velocities genuinely depend on ϕ, and the notation, $v_{r}(\phi ,s,t)$ and $v_{\phi }(\phi ,s,t)$, is natural because the physical motion is no longer axisymmetric. Although swirl generally breaks axisymmetry unless counteracted by viscous or surface tension effects, we continue to assume that the centre-line remains the geometric centroid of each cross section and lies in the *xz*-plane for the purpose of this study.

Since second partial derivatives exist and are continuous, we have

17a $$\begin{aligned}&\begin{aligned} \frac{\partial }{\partial t}\left( \frac{\partial \,{\boldsymbol{r}}_{\text {c}}}{\partial s}\right)&= \frac{\partial }{\partial s}\left( \frac{\partial \,{\boldsymbol{r}}_{\text {c}}}{\partial t}\right) , \end{aligned} \end{aligned}$$

17b $$\begin{aligned}&\begin{aligned} \frac{\partial }{\partial t}\left( \frac{\partial \,{\boldsymbol{r}}_{\text {c}}}{\partial \phi }\right)&= \frac{\partial }{\partial \phi }\left( \frac{\partial \,{\boldsymbol{r}}_{\text {c}}}{\partial t}\right) , \end{aligned} \end{aligned}$$

and when expanded, we obtain kinematic constraints describing the motion of the centre-line

18a $$\begin{aligned}&\begin{aligned}&\frac{\partial \theta }{\partial t}\cos {\phi } = \frac{\partial v_{r}}{\partial s} + \kappa \cos {\phi }\,v_{s}, \end{aligned} \end{aligned}$$

18b $$\begin{aligned}&\begin{aligned}&\frac{\partial \theta }{\partial t}\sin {\phi } = \kappa \sin {\phi }\,v_{s} - \frac{\partial v_{\phi }}{\partial s}, \end{aligned} \end{aligned}$$

18c $$\begin{aligned}&\begin{aligned}&0 = \frac{\partial v_{s}}{\partial s} - \kappa \cos {\phi }\,v_{r} + \kappa \sin {\phi }\,v_{\phi }, \end{aligned} \end{aligned}$$

18d $$\begin{aligned}&\begin{aligned}&0 = \frac{\partial v_{r}}{\partial \phi } - v_{\phi }, \end{aligned} \end{aligned}$$

18e $$\begin{aligned}&\begin{aligned}&0 = \frac{\partial v_{\phi }}{\partial \phi } + v_{r}, \end{aligned} \end{aligned}$$

18f $$\begin{aligned}&\begin{aligned}&0 = \frac{\partial v_{s}}{\partial \phi }, \end{aligned} \end{aligned}$$

with κ as defined in (8c).

#### Governing equations for incompressible fluid flow

In Sect. 2.1.1 we devised a means to describe the geometry and motion of the slender filament with respect to some fixed frame of reference. We must also model the fluid flow within the filament (with respect to the centre-line). Denoting fluid velocity within the filament by ${\boldsymbol{U}} = U_{r}\,{\boldsymbol{e}}_{r} + U_{\phi }\,{\boldsymbol{e}}_{\phi } + U_{s}\,{\boldsymbol{e}}_{s}= (u_{r}+v_{r})\,{\boldsymbol{e}}_{r} + (u_{\phi }+v_{\phi })\,{\boldsymbol{e}}_{\phi } + (u_{s}+v_{s})\,{\boldsymbol{e}}_{s}$, relative to some fixed lab frame, with ${\boldsymbol{u}}$ the fluid velocity relative to the (moving) centre-line and ${\boldsymbol{v}}$ the centre-line velocity, the flow can be characterised via the following governing equations for incompressible fluid flow:

19a $$\begin{aligned}&\begin{aligned} \rho \left( \frac{\partial {\boldsymbol{U}}}{\partial t}+({\boldsymbol{U}}\cdot \boldsymbol{\nabla }){\boldsymbol{U}}\right)&= \boldsymbol{\nabla }\cdot \boldsymbol{\sigma } + \rho \,{\boldsymbol{g}}, \end{aligned} \end{aligned}$$

19b $$\begin{aligned}&\begin{aligned} \boldsymbol{\nabla }\cdot (\rho \,{\boldsymbol{U}})&=0, \end{aligned} \end{aligned}$$

with (19a) *conservation of momentum* and (19b) *conservation of mass*, where $\boldsymbol{\sigma }$ denotes the stress tensor and ${\boldsymbol{g}}$ gravitational acceleration (taken to be $-g\,{\boldsymbol{e}}_{z}$). The constitutive law for the stress tensor of a power-law fluid can be written as

20 $$\begin{aligned} \sigma _{ij} = -p\,\delta _{ij} + \eta (\dot{\gamma })\left( \boldsymbol{\nabla }{\boldsymbol{U}} + (\boldsymbol{\nabla }{\boldsymbol{U}})^{\textrm{T}}\right) _{ij}, \end{aligned}$$

with i,j,k=r,ϕ,s, where *p* denotes the hydrostatic pressure of the fluid, $\delta _{ij}$ the Kronecker delta ($\delta _{ij}=1$ if i=j, and 0 otherwise), $\eta (\dot{\gamma })$ the apparent viscosity as defined in (1), and

21 $$\begin{aligned} \left( \boldsymbol{\nabla }{\boldsymbol{U}}\right) _{ij} = \frac{1}{L_{j}}\frac{\partial U_{i}}{\partial q_{j}} - \frac{U_{j}}{L_{i}L_{j}}\frac{\partial L_{j}}{\partial q_{i}} + \delta _{ij}\sum _{k}\frac{U_{k}}{L_{i}L_{k}}\frac{\partial L_{i}}{\partial q_{k}}. \end{aligned}$$

Via use of scaling factors (12), we expand the coordinate-free system of equations within an arc length coordinate system to generate the *s*-, ϕ-, and *r*-components of (19a) to be

22a $$\begin{aligned}&\begin{aligned}&\rho \left( \frac{\partial }{\partial t}(u_{s}+v_{s}) + (u_{r} + v_{r})\frac{\partial u_{s}}{\partial r} + \frac{(u_{\phi } + v_{\phi })}{r}\frac{\partial u_{s}}{\partial \phi } + \frac{(u_{s}+v_{s})}{L_{s}}\frac{\partial u_{s}}{\partial s} \right. \\&\left. \qquad + \, \frac{\kappa \,(u_{s}+v_{s})}{L_{s}}\left( \sin {\phi }\,u_{\phi } - \cos {\phi }\,u_{r}\right) \right) \\&\quad = \frac{\partial \sigma _{rs}}{\partial r} + \frac{1}{r}\frac{\partial \sigma _{\phi s}}{\partial \phi } + \frac{1}{L_{s}}\frac{\partial \sigma _{ss}}{\partial s} + \frac{\sigma _{rs}}{r} - \frac{2\kappa }{L_{s}}\left( \cos {\phi }\,\sigma _{rs} - \sin {\phi }\,\sigma _{\phi s}\right) + \rho g\cos {\theta }, \end{aligned} \end{aligned}$$

22b $$\begin{aligned}&\begin{aligned}&\rho \left( \frac{\partial }{\partial t}(u_{\phi }+v_{\phi }) + (u_{r}+v_{r})\frac{\partial u_{\phi }}{\partial r} + \frac{(u_{\phi }+v_{\phi })}{r}\frac{\partial u_{\phi }}{\partial \phi } + \frac{(u_{s}+v_{s})}{L_{s}}\frac{\partial u_{\phi }}{\partial s} \right. \\&\left. \qquad + \frac{u_{r}(u_{\phi }+v_{\phi })}{r} - \frac{\sin {\phi }\,(u_{s}+v_{s})}{L_{s}}\left( \kappa \,u_{s} + \frac{\partial \theta }{\partial t}\right) \right) \\&\quad = \frac{\partial \sigma _{r\phi }}{\partial r} + \frac{1}{r}\frac{\partial \sigma _{\phi \phi }}{\partial \phi } + \frac{1}{L_{s}}\frac{\partial \sigma _{s\phi }}{\partial s} + \frac{2\sigma _{r\phi }}{r} + \frac{\kappa }{L_{s}}\left( \sin {\phi }\,(\sigma _{\phi \phi }-\sigma _{ss}) - \cos {\phi }\,\sigma _{r\phi }\right) \\&\qquad + \rho g\sin {\theta }\sin {\phi }, \end{aligned} \end{aligned}$$

22c $$\begin{aligned}&\begin{aligned}&\rho \left( \frac{\partial }{\partial t}(u_{r}+v_{r}) + (u_{r}+v_{r})\frac{\partial u_{r}}{\partial r} + \frac{(u_{\phi }+v_{\phi })}{r}\frac{\partial u_{r}}{\partial \phi } + \frac{(u_{s}+v_{s})}{L_{s}}\frac{\partial u_{r}}{\partial s} \right. \\&\left. \qquad - \frac{u_{\phi }(u_{\phi }+v_{\phi })}{r} + \frac{\cos {\phi }\,(u_{s}+v_{s})}{L_{s}}\left( \kappa \,u_{s} + \frac{\partial \theta }{\partial t}\right) \right) \\&\quad = \frac{\partial \sigma _{rr}}{\partial r} + \frac{1}{r}\frac{\partial \sigma _{\phi r}}{\partial \phi } + \frac{1}{L_{s}}\frac{\partial \sigma _{sr}}{\partial s} + \frac{\sigma _{rr}-\sigma _{\phi \phi }}{r} + \frac{\kappa }{L_{s}}\left( \cos {\phi }\,(\sigma _{ss} - \sigma _{rr}) + \sin {\phi }\,\sigma _{\phi r}\right) \\&\qquad - \rho g\sin {\theta }\cos {\phi }, \end{aligned} \end{aligned}$$

where $\boldsymbol{\sigma }$ has the following components:

23a $$\begin{aligned}&\begin{aligned}&\sigma _{rr} = -p + 2K\dot{\gamma }^{n-1}\frac{\partial u_{r}}{\partial r}, \end{aligned} \end{aligned}$$

23b $$\begin{aligned}&\begin{aligned}&\sigma _{\phi \phi } = -p + \frac{2K\dot{\gamma }^{n-1}}{r}\left( \frac{\partial u_{\phi }}{\partial \phi } + u_{r}\right) , \end{aligned} \end{aligned}$$

23c $$\begin{aligned}&\begin{aligned}&\sigma _{ss} = -p + \frac{2K\dot{\gamma }^{n-1}}{L_{s}}\left( \frac{\partial u_{s}}{\partial s} + \kappa \,(\sin {\phi }\,u_{\phi } - \cos {\phi }\,u_{r})\right) , \end{aligned} \end{aligned}$$

23d $$\begin{aligned}&\begin{aligned}&\sigma _{r\phi } = \sigma _{\phi r} = K\dot{\gamma }^{n-1}\left( \frac{\partial u_{\phi }}{\partial r} + \frac{1}{r}\left( \frac{\partial u_{r}}{\partial \phi } - u_{\phi }\right) \right) , \end{aligned} \end{aligned}$$

23e $$\begin{aligned}&\begin{aligned}&\sigma _{\phi s} = \sigma _{s\phi } = K\dot{\gamma }^{n-1}\left( \frac{1}{r}\frac{\partial u_{s}}{\partial \phi } + \frac{1}{L_{s}}\left( \frac{\partial u_{\phi }}{\partial s} - \sin {\phi }\left( \kappa \,u_{s} + \frac{\partial \theta }{\partial t}\right) \right) \right) , \end{aligned} \end{aligned}$$

23f $$\begin{aligned}&\begin{aligned}&\sigma _{rs} = \sigma _{sr} = K\dot{\gamma }^{n-1}\left( \frac{\partial u_{s}}{\partial r} + \frac{1}{L_{s}}\left( \frac{\partial u_{r}}{\partial s} + \cos {\phi }\left( \kappa \,u_{s} + \frac{\partial \theta }{\partial t}\right) \right) \right) . \end{aligned} \end{aligned}$$

Conservation of mass (19b) is given by

24 $$\begin{aligned} \rho \left( \frac{u_{r}}{r} + \frac{\partial u_{r}}{\partial r} + \frac{1}{r}\frac{\partial u_{\phi }}{\partial \phi } + \frac{1}{L_{s}}\frac{\partial u_{s}}{\partial s} - \frac{\kappa }{L_{s}}\left( \cos {\phi }\,u_{r} - \sin {\phi }\,u_{\phi }\right) \right) = 0. \end{aligned}$$

The shear rate is determined via

25 $$\begin{aligned} \dot{\gamma }^{2} = \frac{1}{2}\boldsymbol{\dot{\gamma }}\cdot \boldsymbol{\dot{\gamma }} = \frac{1}{2}\sum _{i,j}\left( \boldsymbol{\nabla }{\boldsymbol{U}} + (\boldsymbol{\nabla }{\boldsymbol{U}})^T\right) _{ij}\left( \boldsymbol{\nabla }{\boldsymbol{U}} + (\boldsymbol{\nabla }{\boldsymbol{U}})^{\textrm{T}}\right) _{ji}, \end{aligned}$$

where $\boldsymbol{\dot{\gamma }}$ denotes the shear rate tensor, as introduced by Morozov and Spagnolie [22] in the context of modelling complex fluids. Expanding (25) within an arc length coordinate system, we obtain

26 $$\begin{aligned} \begin{aligned} \dot{\gamma }^{2}&= 2\left( \frac{\partial u_{r}}{\partial r}\right) ^{2} + \frac{2}{r^{2}}\left( \frac{\partial u_{\phi }}{\partial \phi } + u_{r}\right) ^{2} + \frac{2}{L_{s}^{2}}\left( \frac{\partial u_{s}}{\partial s} + \kappa \left( \sin {\phi }\,u_{\phi } - \cos {\phi }\,u_{r}\right) \right) ^{2} \\&\hspace{0.4cm} + \frac{1}{2}\left( \frac{\partial u_{\phi }}{\partial r} + \frac{1}{r}\left( \frac{\partial u_{r}}{\partial \phi } - u_{\phi }\right) \right) ^{2} \\&\hspace{0.4cm} + \frac{1}{2}\left( \frac{\partial u_{s}}{\partial r} + \frac{1}{L_{s}}\left( \frac{\partial u_{r}}{\partial s} + \cos {\phi }\left( \kappa \, u_{s} + \frac{\partial \theta }{\partial t}\right) \right) \right) ^{2} \\&\hspace{0.4cm} + \frac{1}{2}\left( \frac{1}{r}\frac{\partial u_{s}}{\partial \phi } + \frac{1}{L_{s}}\left( \frac{\partial u_{\phi }}{\partial s} - \sin {\phi }\left( \kappa \, u_{s} + \frac{\partial \theta }{\partial t}\right) \right) \right) ^{2}. \end{aligned} \end{aligned}$$

Taking the positive square root of (26) and substituting into (1), we arrive at an expression for the apparent viscosity of a power-law fluid.

With the centre-line denoted as in (9), an arbitrary point in the filament cross section is represented by ${\boldsymbol{r}}(r,\phi ,s,t) = {\boldsymbol{r}}_{\text {c}}(s,t) + r\,{\boldsymbol{e}}_{r}$ [corresponding with (11)], where ${\boldsymbol{r}}_{\text {b}}(\phi ,s,t) = {\boldsymbol{r}}_{\text {c}}(s,t) + R(\phi , s, t)\,{\boldsymbol{e}}_{r}$ captures points on the filament boundary. Hence, the velocity of the fluid relative to an arbitrary point ${\boldsymbol{r}}$ in the cross section is given by ${\boldsymbol{U}} - \partial ({\boldsymbol{r}}_{\text {c}} + r\,{\boldsymbol{e}}_{r})/\partial t$. Taking the material derivative along the relative flow and evaluating at the boundary

27 $$\begin{aligned} \frac{\text {D}}{\text {D}t}(R-r) = \left[ \frac{\partial }{\partial t}(R-r) + \left( \left( {\boldsymbol{U}} - \frac{\partial }{\partial t}({\boldsymbol{r}}_{\text {c}} + r\,{\boldsymbol{e}}_{r})\right) \cdot \boldsymbol{\nabla }\right) (R-r)\right] _{r=R(\phi , s, t)} = 0, \end{aligned}$$

we obtain a kinematic condition describing the motion of fluid particles along the free surface

28 $$\begin{aligned} u_{r}(r, \phi , s, t) = \frac{\partial R}{\partial t} + \frac{u_{\phi }}{r}\frac{\partial R}{\partial \phi } + \frac{1}{L_{s}}\frac{\partial R}{\partial s}\left( u_{s} + r\,\frac{\partial \theta }{\partial t}\cos {\phi }\right) , \quad \text {at} \quad r=R(\phi , s, t), \end{aligned}$$

i.e. if a fluid particle starts on the boundary it will remain on the boundary. We also assume surface tension negligible and impose no-stress condition

29 $$\begin{aligned} \boldsymbol{\sigma }\cdot {\boldsymbol{n}} = {\boldsymbol{0}}, \end{aligned}$$

with

30 $$\begin{aligned} \begin{aligned} {\boldsymbol{n}}&= \left| \frac{\partial {\boldsymbol{r}}_{\text {b}}}{\partial \phi }\times \frac{\partial {\boldsymbol{r}}_{\text {b}}}{\partial s}\right| ^{-1}\, \frac{\partial {\boldsymbol{r}}_{\text {b}}}{\partial \phi }\times \frac{\partial {\boldsymbol{r}}_{\text {b}}}{\partial s} \\&= \frac{1}{\sqrt{\varLambda }}\left( R\tilde{L}_{s}\,{\boldsymbol{e}}_{r} -\frac{\partial R}{\partial \phi }\tilde{L}_{s}\,{\boldsymbol{e}}_{\phi } -R\frac{\partial R}{\partial s}\,{\boldsymbol{e}}_{s}\right) , \end{aligned} \end{aligned}$$

the outward-pointing unit normal to the surface, with $\tilde{L}_{s}=1-R\,\kappa \cos {\phi }$, and

31 $$\begin{aligned} \varLambda = R^{2}\left( \tilde{L}_{s}^{2}+\left( \frac{\partial R}{\partial s}\right) ^{2}\right) +\left( \frac{\partial R}{\partial \phi }\right) ^{2}\tilde{L}_{s}^{2}. \end{aligned}$$

Equations (22a)–(24) are also subject to the following initial conditions:

32 $$\begin{aligned} u_{i}(r, \phi , s, t) = 0, \quad \text {for} \quad i=r, \phi , \quad \text {at} \quad r=0, \end{aligned}$$

as well as those imposed by the pneumatic-driven bioprinter set-up itself

33a $$\begin{aligned}&\begin{aligned}&R(\phi , s, t) = R_{\text {N}}, \qquad \text {at} \qquad s=0, \end{aligned} \end{aligned}$$

33b $$\begin{aligned}&\begin{aligned}&u_{s}(r,\phi , s, t) = u_{\text {E}}, \quad \quad \text {at} \qquad s=0, \end{aligned} \end{aligned}$$

33c $$\begin{aligned}&\begin{aligned}&u_{\phi }(r,\phi , s, t) = 0, \qquad \text {at} \qquad s=0, \end{aligned} \end{aligned}$$

with (32) imposing axisymmetry and a purely axial fluid velocity along the centre-line (i.e. treating the centre-line as a line of symmetry), (33a) setting the initial filament radius to nozzle tip radius, $R_{\text {N}}$, (33b) setting the initial axial fluid velocity to the extrusion speed from the nozzle tip, $u_{\text {E}}$, and (33c) implying a pneumatic-driven set-up, where there involves no initial rotation of the fluid.

Whether the build platform travel speed guides the dragging of the filament along the build platform or the nozzle travel speed, the definition of the rate of fluid flow through an arbitrary filament cross section on the build platform remains the same. Therefore, we impose a relative axial fluid velocity on the build platform

34 $$\begin{aligned} u_{s}(r,\phi , s, t) = U_{\text {N}}, \quad \text {at} \quad s=L(t), \end{aligned}$$

where the orientation of the filament centre-line is

35 $$\begin{aligned} \theta (s,t) = \frac{\pi }{2}, \quad \text {at} \quad s=L(t). \end{aligned}$$

Since the filament cross section is periodic in ϕ, we must also have that the pressure and fluid velocity at ϕ=0 are the same as that at $\phi =2\pi$, i.e.

36 $$\begin{aligned}&p(r, 0, s, t) \quad = p(r, 2\pi , s, t), \nonumber \\&u_{i}(r, 0, s, t) \quad = u_{i}(r, 2\pi , s, t), \qquad \text {for} \quad i =r, \phi , s, \nonumber \\&\frac{\partial u_{i}}{\partial j}(r, 0, s, t) = \frac{\partial u_{i}}{\partial j}(r, 2\pi , s, t), \quad \text {for} \quad i,j =r, \phi , s. \end{aligned}$$

As such, we have generated eight Eqs. (7a), (7b), (10), (22a)–(24), and (28) subject to boundary and initial conditions (29)–(36) and satisfied by seven unknowns describing the extensional flow: the filament radius, R(ϕ,s,t); the orientation of the filament centre-line, θ(s,t); the hydrostatic pressure of the fluid, p(r,ϕ,s,t); the fluid velocity with respect to the (moving) centre-line, $u_{i}(r,\phi ,s,t)$ (for i=r,ϕ,s); and the length of the filament, *L*(*t*). The apparent overdetermination arises because the centre-line has been constrained to lie in the *xz*-plane, which reduces the number of centre-line orientation variables from two independent angles to a single θ(s,t). With this planar constraint in place, the centroid relations (7) are not independent, and the redundancy becomes apparent during the asymptotic reduction (see Sect. 2.1.5).

#### Non-dimensionalisation

Introducing characteristic scales appropriate for pneumatic extrusion, given by

37a $$\begin{aligned}&\begin{aligned}&s = Ls^{*}, \quad r=\varepsilon ^{n} Lr^{*}, \quad \kappa = \frac{\kappa ^{*}}{L}, \quad R=R_{\text {N}}R^{*}, \quad t=\frac{L}{\bar{u}_{\text {E}}}t^{*}, \\&p=K\left( \frac{\bar{u}_{\text {E}}}{L}\right) ^{n}\,p^{*}, \end{aligned} \end{aligned}$$

37b $$\begin{aligned}&\begin{aligned}&u_{i} = {\left\{ \begin{array}{ll} \bar{u}_{\text {E}}\,u_{i}^{*}, &  \text {if}\, i = s, \\ \varepsilon ^{n}\,\bar{u}_{\text {E}}\,u_{i}^{*}, &  \text {otherwise}, \end{array}\right. } \end{aligned} \end{aligned}$$

37c $$\begin{aligned}&\begin{aligned}&v_{i} = \bar{u}_{\text {E}}\,v_{i}^{*}, \quad \text {for} \quad i = r, \phi , s, \end{aligned} \end{aligned}$$

37d $$\begin{aligned}&\begin{aligned}&\sigma _{ij} = {\left\{ \begin{array}{ll} K\left( \frac{\bar{u}_{\text {E}}}{\varepsilon ^{n} L}\right) ^{n}\sigma _{ij}^{*}, &  \text {if} \, (i,j)=(\phi ,s), (s,\phi ), (r,s) \; \text {or} \; (s,r), \\ K\left( \frac{\bar{u}_{\text {E}}}{L}\right) ^{n}\sigma _{ij}^{*}, &  \text {otherwise}, \end{array}\right. } \end{aligned} \end{aligned}$$

37e $$\begin{aligned}&\begin{aligned}&\dot{\gamma } = \frac{\bar{u}_{\text {E}}}{L}\dot{\gamma }^{*}, \end{aligned} \end{aligned}$$

37f $$\begin{aligned}&\begin{aligned}&n_{i} = {\left\{ \begin{array}{ll} n_{i}^{*}, &  \text {if} \, i = r,\phi , \\ \varepsilon ^{n}\,n_{i}^{*}, &  \text {if} \, i=s, \end{array}\right. } \end{aligned} \end{aligned}$$

where * denotes the dimensionless variables. The dimensionless formulation is governed by the flow behaviour index *n*, the slenderness parameter

38 $$\begin{aligned} \varepsilon ^{n}=\frac{R_{\text {N}}}{L}, \end{aligned}$$

as well as a generalised Reynolds number for a power-law fluid, and the Froude number

39 $$\begin{aligned} \text {Re}_{\text {PL}} = \frac{\rho \,(\bar{u}_{\text {E}})^{2-n}L^{n}}{K} \quad \text {and} \quad \text {Fr} = \frac{\bar{u}_{\text {E}}}{\sqrt{gL}}. \end{aligned}$$

Here, *n* characterises the degree of shear-thinning, $\varepsilon ^{n}$ characterises filament slenderness, while $\text {Re}_{\text {PL}}$ and $\text {Fr}$ describe the relative importance of inertial, viscous, and gravitational effects. Experimentally, $\varepsilon ^{n} = {\mathcal {O}}(10^{-2})$ when $0.40\le n \le 0.65$. Based on prior experimental observation, Paxton et al. [10] specify that extrusion speeds equal to or slightly larger than the nozzle travel speed, $U_{\text {N}}$, are acceptable, justifying our choice of velocity scale, ${\boldsymbol{v}}$, given by (37c). These dimensionless equations form the basis for the asymptotic analysis presented in the next section. Full details of the non-dimensionalisation, including the complete dimensionless governing equations and boundary conditions, are provided in Supporting Information.

#### Slender-body asymptotics

Exploiting the slenderness of the filament geometry, we perform an asymptotic expansion in powers of $\varepsilon ^{n}$

40 $$\begin{aligned} \begin{aligned}&u_{i} \sim u_{i}^{(0)} + \varepsilon ^{n}\,u_{i}^{(1)} + \cdots , \quad v_{i} \sim v_{i}^{(0)} + \varepsilon ^{n}\,v_{i}^{(1)} + \cdots , \quad p \sim p^{(0)} + \varepsilon ^{n}\,p^{(1)} +\cdots , \\&R \sim R^{(0)} + \varepsilon ^{n}R^{(1)} + \cdots , \quad \theta \sim \theta ^{(0)} + \varepsilon ^{n}\,\theta ^{(1)} + \cdots , \quad \kappa \sim \kappa ^{(0)} + \varepsilon ^{n}\kappa ^{(1)} + \cdots , \\&L \sim L^{(0)} + \varepsilon ^{n} L^{(1)} +\cdots , \end{aligned} \end{aligned}$$

where we have dropped the stars for convenience and i=r,ϕ,s. Since 0<n<1 for shear-thinning materials, it follows that $\varepsilon<\varepsilon ^{n}<1$. Paxton et al. [10] reported a flow behaviour index, *n*, equal to 0.552 for Nivea Crème, consistent with its previously characterised shear-thinning behaviour. Having restricted asymptotic analysis to moderately shear-thinning materials (i.e. $0.40\le n \le 0.65$), and identified that $\varepsilon ^{n} = {\mathcal {O}}(10^{-2})$ (as in (38)), we obtain $\varepsilon = {\mathcal {O}}(10^{-4})$. This small parameter supports the applicability of an asymptotic analysis. Furthermore, we assume extrusion speed, $u_{\text {E}}$, to be constant and uniform at leading order (i.e. *plug flow* within the filament), that is

41 $$\begin{aligned} u_{\text {E}} = \bar{u}_{\text {E}}\left( 1 + \varepsilon ^{n}\, u_{\text {E}}^{(1)} + \cdots \right) , \end{aligned}$$

where $u_{\text {E}}^{(k)}$ (for $k\in {\mathbb {N}}$) are unknown functions of *r* and ϕ. We proceed to substitute (40) and (41) into the dimensionless forms of Eqs. (10) and (22a)–(36) before collecting powers of $\varepsilon ^{n}$ to obtain relations at certain orders of magnitude.

We begin by assuming all leading-order variables independent of ϕ. Considering conservation of momentum: (22a) yields

42a $$\begin{aligned}&\begin{aligned} 0 =&\, \frac{1}{r}\frac{\partial }{\partial r}\left( r\sigma _{rs}^{(0)}\right) , \end{aligned} \end{aligned}$$

42b $$\begin{aligned}&\begin{aligned} 0 =&\, \frac{1}{r}\frac{\partial }{\partial r}\left( r\sigma _{rs}^{(1)}\right) + \frac{1}{r}\frac{\partial \sigma _{\phi s}^{(1)}}{\partial \phi } - 2\,\kappa ^{(0)}\cos {\phi }\,\sigma _{rs}^{(0)}, \end{aligned} \end{aligned}$$

42c $$\begin{aligned}&\begin{aligned} 0 =&\, \frac{1}{r}\frac{\partial }{\partial r}\left( r\sigma _{rs}^{(2)}\right) + \frac{1}{r}\frac{\partial \sigma _{\phi s}^{(2)}}{\partial \phi } + \frac{\partial \sigma _{ss}^{(0)}}{\partial s} - 2\kappa ^{(0)}\left( \cos {\phi }\,\sigma _{rs}^{(1)} - \sin {\phi }\,\sigma _{\phi s}^{(1)}\right) \\&\quad - 2\left( r\left( \kappa ^{(0)}\right) ^{2}\cos {\phi } + \kappa ^{(1)}\right) \cos {\phi }\,\sigma _{rs}^{(0)} + \frac{\text {Re}_{\text {PL}}}{\text {Fr}^{2}}\cos {\theta ^{(0)}}, \end{aligned} \end{aligned}$$

at leading order, first order, and second order; (22b) yields

43a $$\begin{aligned}&\begin{aligned} 0 =&\, \frac{1}{r^{2}}\frac{\partial }{\partial r}\left( r^{2}\sigma _{r\phi }^{(0)}\right) + \frac{1}{r}\frac{\partial \sigma _{\phi \phi }^{(0)}}{\partial \phi }, \end{aligned} \end{aligned}$$

43b $$\begin{aligned}&\begin{aligned} 0 =&\,\frac{1}{r^{2}}\frac{\partial }{\partial r}\left( r^{2}\sigma _{r\phi }^{(1)}\right) + \frac{1}{r}\frac{\partial \sigma _{\phi \phi }^{(1)}}{\partial \phi } + \frac{\partial \sigma _{s\phi }^{(1)}}{\partial s} + \kappa ^{(0)}\left( \sin {\phi }\left( \sigma _{\phi \phi }^{(0)} - \sigma _{ss}^{(0)}\right) - \cos {\phi }\,\sigma _{r\phi }^{(0)}\right) \\&\quad + \frac{\text {Re}_{\text {PL}}}{\text {Fr}^{2}}\sin {\theta ^{(0)}}\sin {\phi }, \end{aligned} \end{aligned}$$

at leading order and first order; and (22c) yields

44a $$\begin{aligned}&\begin{aligned} 0 =&\, \frac{1}{r}\frac{\partial }{\partial r}\left( r\sigma _{rr}^{(0)}\right) + \frac{1}{r}\frac{\partial \sigma _{\phi r}^{(0)}}{\partial \phi } + \frac{\partial \sigma _{sr}^{(0)}}{\partial s} - \frac{\sigma _{\phi \phi }^{(0)}}{r}, \end{aligned} \end{aligned}$$

44b $$\begin{aligned}&\begin{aligned} 0 =&\, \frac{1}{r}\frac{\partial }{\partial r}\left( r\sigma _{rr}^{(1)}\right) + \frac{1}{r}\frac{\partial \sigma _{\phi r}^{(1)}}{\partial \phi } + \frac{\partial \sigma _{sr}^{(1)}}{\partial s} + r\kappa ^{(0)}\cos {\phi }\,\frac{\partial \sigma _{sr}^{(0)}}{\partial s} - \frac{\sigma _{\phi \phi }^{(1)}}{r} \\&\quad + \kappa ^{(0)}\left( \cos {\phi }\left( \sigma _{ss}^{(0)} - \sigma _{rr}^{(0)}\right) - \sin {\phi }\,\sigma _{\phi r}^{(0)}\right) - \frac{\text {Re}_{\text {PL}}}{\text {Fr}^{2}}\sin {\theta ^{(0)}}\cos {\phi }, \end{aligned} \end{aligned}$$

at leading order and first order, where $\text {Re}_{\text {PL}}$ and $\text {Fr}$ are the generalised Reynolds and Froude numbers introduced in Sect. 2.1.3. Based on work of Paxton et al. [10] and Tu et al. [23], both $\text {Re}_{\text {PL}}$ and $\text {Fr}^{2}$ are small, taking values of order $10^{-2n^{2}+3n-6}\sim 10^{-5}\ll 1$ (when $0.40\le n \le 0.65$) and $10^{-5}\ll 1$, respectively, making their ratio approximately leading order. With $\text {Re}_{\text {PL}}$ small, we remark that we are considering a *viscous-dominated regime* with negligible inertial forces.

Furthermore, at leading order, conservation of mass (24) provides

45 $$\begin{aligned} \frac{1}{r}\frac{\partial }{\partial r}\left( r\,u_{r}^{(0)}\right) + \frac{\partial u_{s}^{(0)}}{\partial s} = 0, \end{aligned}$$

while (10) provides

46 $$\begin{aligned} \int _0^{L^{(0)}/L} \cos {\theta ^{(0)}}(s,t)\,\text {d}s = \frac{H}{L}. \end{aligned}$$

Centroid integrals, (7a) and (7b), provide

47a $$\begin{aligned}&\begin{aligned} 0 =&\int _{0}^{2\pi } \left( R^{(0)}\right) ^{3}\cos {\phi } \;\text {d}\phi , \end{aligned} \end{aligned}$$

47b $$\begin{aligned}&\begin{aligned} 0 =&\int _{0}^{2\pi } \left( R^{(0)}\right) ^{2}R^{(1)}\cos {\phi } \;\text {d}\phi , \end{aligned} \end{aligned}$$

47c $$\begin{aligned}&\begin{aligned} 0 =&\int _{0}^{2\pi } R^{(0)}\left( R^{(0)}R^{(2)} + \left( R^{(1)}\right) ^{2}\right) \cos {\phi } \;\text {d}\phi , \end{aligned} \end{aligned}$$

47d $$\begin{aligned}&\begin{aligned} 0 =&\int _{0}^{2\pi } \left( R^{(0)}\right) ^{3}\sin {\phi } \;\text {d}\phi , \end{aligned} \end{aligned}$$

47e $$\begin{aligned}&\begin{aligned} 0 =&\int _{0}^{2\pi } \left( R^{(0)}\right) ^{2}R^{(1)}\sin {\phi } \;\text {d}\phi , \end{aligned} \end{aligned}$$

47f $$\begin{aligned}&\begin{aligned} 0 =&\int _{0}^{2\pi } R^{(0)}\left( R^{(0)}R^{(2)} + \left( R^{(1)}\right) ^{2}\right) \sin {\phi } \;\text {d}\phi , \end{aligned} \end{aligned}$$

at leading order, first order, and second order. This system of equations is then subject to the leading-order kinematic boundary condition

48 $$\begin{aligned} \left[ u_{r}^{(0)}\right] _{r=R^{(0)}} = \left[ \frac{\partial R^{(0)}}{\partial t} + \frac{\partial R^{(0)}}{\partial s}u_{s}^{(0)}\right] _{r=R^{(0)}}, \end{aligned}$$

together with the no-stress condition at various orders, given by

49a $$\begin{aligned}&\begin{aligned} 0 =&\left[ R^{(0)}\sigma _{rr}^{(0)} - R^{(0)}\frac{\partial R^{(0)}}{\partial s}\sigma _{rs}^{(0)}\right] _{r=R^{(0)}}, \end{aligned} \end{aligned}$$

49b $$\begin{aligned}&\begin{aligned} 0 =&\left[ R^{(0)}\sigma _{rr}^{(1)} + \left( R^{(1)} -\left( R^{(0)}\right) ^{2}\kappa ^{(0)}\cos {\phi }\right) \sigma _{rr}^{(0)} - \frac{\partial R^{(1)}}{\partial \phi }\sigma _{r \phi }^{(0)} \right. \\&\quad \left. -R^{(0)}\frac{\partial R^{(0)}}{\partial s}\sigma _{rs}^{(1)} - \left( R^{(0)}\frac{\partial R^{(1)}}{\partial s} + R^{(1)}\frac{\partial R^{(0)}}{\partial s}\right) \sigma _{rs}^{(0)} \right. \\&\quad \left. + R^{(1)}\frac{\partial }{\partial r}\left( R^{(0)}\sigma _{rr}^{(0)} - R^{(0)}\frac{\partial R^{(0)}}{\partial s}\sigma _{rs}^{(0)}\right) \right] _{r=R^{(0)}}, \end{aligned} \end{aligned}$$

49c $$\begin{aligned}&\begin{aligned} 0 =&\left[ R^{(0)}\sigma _{\phi r}^{(0)}\right] _{r=R^{(0)}}, \end{aligned} \end{aligned}$$

49d $$\begin{aligned}&\begin{aligned} 0 =&\left[ R^{(0)}\sigma _{\phi r}^{(1)} + \left( R^{(1)} -\left( R^{(0)}\right) ^{2}\kappa ^{(0)}\cos {\phi }\right) \sigma _{\phi r}^{(0)} - \frac{\partial R^{(1)}}{\partial \phi }\sigma _{\phi \phi }^{(0)} \right. \\&\quad \left. -R^{(0)}\frac{\partial R^{(0)}}{\partial s}\sigma _{\phi s}^{(1)} + R^{(1)}R^{(0)}\frac{\partial \sigma _{\phi r}^{(0)}}{\partial r}\right] _{r=R^{(0)}}, \end{aligned} \end{aligned}$$

49e $$\begin{aligned}&\begin{aligned} 0 =&\left[ R^{(0)}\sigma _{sr}^{(0)}\right] _{r=R^{(0)}}, \end{aligned} \end{aligned}$$

49f $$\begin{aligned}&\begin{aligned} 0 =&\left[ R^{(0)}\sigma _{sr}^{(1)} + \left( R^{(1)} -\left( R^{(0)}\right) ^{2}\kappa ^{(0)}\cos {\phi }\right) \sigma _{sr}^{(0)} + R^{(1)}R^{(0)}\frac{\partial \sigma _{sr}^{(0)}}{\partial r}\right] _{r=R^{(0)}}, \end{aligned} \end{aligned}$$

49g $$\begin{aligned}&\begin{aligned} 0 =&\left[ R^{(0)}\sigma _{sr}^{(2)} + \left( R^{(1)} -\left( R^{(0)}\right) ^{2}\kappa ^{(0)}\cos {\phi }\right) \sigma _{sr}^{(1)} - \frac{\partial R^{(1)}}{\partial \phi }\sigma _{s\phi }^{(1)} \right. \\&\quad \left. - R^{(0)}\frac{\partial R^{(0)}}{\partial s}\sigma _{ss}^{(0)} + \left( R^{(2)} -R^{(0)}\cos {\phi }\left( 2R^{(1)}\kappa ^{(0)} + R^{(0)}\kappa ^{(1)}\right) \right) \sigma _{sr}^{(0)} \right. \\&\quad \left. + R^{(2)}R^{(0)}\frac{\partial \sigma _{sr}^{(0)}}{\partial r} + R^{(1)}\frac{\partial }{\partial r}\left( R^{(0)}\sigma _{sr}^{(1)} + \left( R^{(1)} -\left( R^{(0)}\right) ^{2}\kappa ^{(0)}\cos {\phi }\right) \sigma _{sr}^{(0)}\right) \right. \\&\quad \left. + \frac{1}{2}R^{(0)}\left( R^{(1)}\right) ^{2}\frac{\partial ^{2}\sigma _{sr}^{(0)}}{\partial r^{2}}\right] _{r=R^{(0)}}, \end{aligned} \end{aligned}$$

with (49a) and (49b) the *r*-component at leading order and first order, (49c) and (49d) the ϕ-component at leading order and first order, and (49e)–(49g) the *s*-component at leading order, first order, and second order, with

50 $$\begin{aligned} \left( \dot{\gamma }^{n-1}\right) ^{(0)} = \left( 3\left( \frac{\partial u_{s}^{(0)}}{\partial s}\right) ^{2}\right) ^{\frac{n-1}{2}}, \end{aligned}$$

and $\sigma _{ij}^{(k)}$ (for i,j=r,ϕ,s, and k=0,1,2) provided in Supplementary Information. The system of equations is also subject to conditions

51a $$\begin{aligned}&\begin{aligned}&u_{i}^{(0)}(r, \phi , s, t) = 0, \quad \quad \text {for} \quad i = r, \phi , \quad \text {at} \quad r=0, \end{aligned} \end{aligned}$$

51b $$\begin{aligned}&\begin{aligned}&R^{(0)}(\phi , s, t) = 1, \qquad \qquad \!\!\! \text {at} \quad s=0, \end{aligned} \end{aligned}$$

51c $$\begin{aligned}&\begin{aligned}&u_{s}^{(0)}(r, s, t) = 1, \qquad \qquad \text {at} \quad s=0, \end{aligned} \end{aligned}$$

51d $$\begin{aligned}&\begin{aligned}&u_{\phi }^{(0)}(r, s, t) = 0, \qquad \qquad \text {at} \quad s=0, \end{aligned} \end{aligned}$$

51e $$\begin{aligned}&\begin{aligned}&u_{s}^{(0)}(r, s, t) = \frac{U_{\text {N}}}{\bar{u}_{\text {E}}}, \qquad \quad \text {at} \quad s=\frac{L^{(0)}(t)}{L}, \end{aligned} \end{aligned}$$

51f $$\begin{aligned}&\begin{aligned}&\theta ^{(0)}(s, t) = \frac{\pi }{2}, \qquad \qquad \quad \text {at} \quad \quad s=\frac{L^{(0)}(t)}{L}, \end{aligned} \end{aligned}$$

as well as, periodicity conditions

52a $$\begin{aligned}&\begin{aligned}&p^{(k)}(r, 0, s, t) = p^{(k)}(r, 2\pi , s, t),\quad \text {for} \quad k = 0, 1, 2, \end{aligned} \end{aligned}$$

52b $$\begin{aligned}&\begin{aligned}&u_{i}^{(k)}(r, 0, s, t) = u_{i}^{(k)}(r, 2\pi , s, t), \quad \text {for} \quad i = r, \phi , s, \quad \text {and} \quad k = 0, 1, 2, \end{aligned} \end{aligned}$$

52c $$\begin{aligned}&\begin{aligned}&\frac{\partial u_{i}^{(k)}}{\partial j}(r, 0, s, t) = \frac{\partial u_{i}^{(k)}}{\partial j}(r, 2\pi , s, t),\quad \text {for} \quad i,j = r, \phi , s, \quad \text {and} \quad k = 0, 1, 2. \end{aligned} \end{aligned}$$

To close the system at leading order, it was necessary to expand equations to ${\mathcal {O}}(\varepsilon ^{2n}$). As such, we have generated 16 Eqs. (42a)–(44b), (45)–(48) subject to boundary and initial conditions (49a)–(52c) and satisfied by fifteen unknowns describing the extensional flow: the leading-, first-, and second-order filament radius, $R^{(k)}$ (k=0,1,2); the leading- and first-order orientation of the filament centre-line, $\theta ^{(k)}$ (k=0,1); the leading- and first-order hydrostatic pressure of the fluid, $p^{(k)}$ (k=0,1); the leading- and first-order fluid velocity with respect to the (moving) centre-line, $u_{i}^{(k)}$ (i=r,ϕ,s, k=0,1); the second-order axial fluid velocity with respect to the (moving) centre-line, $u_{s}^{(2)}$; and the length of the filament, $L^{(0)}$. As before, the apparent overdetermination reflects geometric constraints already imposed on the centre-line representation, resulting in dependent relations that become evident during the asymptotic reduction presented in the next section.

#### Leading-order steady-state system

The set-up presented in Fig. 1 involves a constant nozzle travel speed, $U_{\text {N}}$, and a stationary build platform which does not vary with time, and therefore, we consider system (42a)–(52c) at *steady-state*. In this state, $L^{(0)}(t)$ is assumed to be a typical filament length, *L*, such that $L^{(0)}(t)/L\sim 1$, and centre-line velocity components, $v_{i}^{(0)}$ (for i=r,ϕ,s), are given by

53a $$\begin{aligned}&\begin{aligned} v_{r}^{(0)}(\phi ,s)&= -\frac{U_{\text {N}}}{\bar{u}_{\text {E}}}\cos {\theta ^{(0)}}\cos {\phi }, \end{aligned} \end{aligned}$$

53b $$\begin{aligned}&\begin{aligned} v_{\phi }^{(0)}(\phi ,s)&= \frac{U_{\text {N}}}{\bar{u}_{\text {E}}}\cos {\theta ^{(0)}}\sin {\phi }, \end{aligned} \end{aligned}$$

53c $$\begin{aligned}&\begin{aligned} v_{s}^{(0)}(s)&= - \frac{U_{\text {N}}}{\bar{u}_{\text {E}}}\sin {\theta ^{(0)}}. \end{aligned} \end{aligned}$$

We begin by integrating (42a) with respect to *r* across an arbitrary cross-sectional area for $r<R^{(0)}(s)$. In doing so, we obtain the leading-order axial fluid velocity with respect to the centre-line, $u_{s}^{(0)}=u_{s}^{(0)}(s)$. Since $u_{s}^{(0)}$ is independent of *r*, we must have boundary condition (49e) hold for all *r* across the cross section, i.e. the axial fluid velocity is uniform across the filament cross section: we are observing *extensional flow*.

The transverse fluid velocity with respect to the centre-line, $u_{r}^{(0)}$, can be determined in a similar way: integrating (45) with respect to *r* across an arbitrary cross-sectional area for $r<R^{(0)}(s)$ generates

54 $$\begin{aligned} u_{r}^{(0)} = -\frac{r}{2}\frac{\text {d}u_{s}^{(0)}}{\text {d}s}, \end{aligned}$$

and, for $r=R^{(0)}(s)$, a conservation of mass equation for the filament cross-sectional area

55 $$\begin{aligned} \frac{\text {d}}{\text {d}s}\left( \pi \left( R^{(0)}\right) ^{2}u_{s}^{(0)}\right) = 0, \end{aligned}$$

having applied boundary condition (48). From (55) we further deduce

56 $$\begin{aligned} u_{s}^{(0)} = \frac{1}{\left( R^{(0)}\right) ^{2}}, \end{aligned}$$

where we applied initial conditions (51b) and (51c).

Likewise, the leading-order azimuthal fluid velocity with respect to the centre-line, $u_{\phi }^{(0)}$, can be obtained by solving the second-order partial differential equation given by

57 $$\begin{aligned} \frac{\partial }{\partial r}\left( \left( \dot{\gamma }^{n-1}\right) ^{(0)}\left( \frac{\partial u_{\phi }^{(0)}}{\partial r} - \frac{u_{\phi }^{(0)}}{r}\right) \right) +\frac{2}{r}\left( \dot{\gamma }^{n-1}\right) ^{(0)}\left( \frac{\partial u_{\phi }^{(0)}}{\partial r} - \frac{u_{\phi }^{(0)}}{r}\right) = 0. \end{aligned}$$

Assuming a general solution of the form

58 $$\begin{aligned} u_{\phi }^{(0)} = c_{1}(s)\,r + \frac{c_{2}(s)}{r}, \end{aligned}$$

we obtain $u_{\phi }^{(0)}=0$, since the azimuthal velocity must be finite at r=0 and, with no change in *s* in (43a), initial condition (51d) must hold along the filament.

Finally, substituting $u_{r}^{(0)}$ and $u_{s}^{(0)}$ into (44a) yields that the leading-order hydrostatic pressure of the fluid, $p^{(0)}$, is independent of *r*. Using boundary condition (49a) we find

59 $$\begin{aligned} p^{(0)} = -\left( \dot{\gamma }^{n-1}\right) ^{(0)}\frac{\text {d}u_{s}^{(0)}}{\text {d}s}, \quad \text {at} \quad r=R^{(0)}(s), \end{aligned}$$

and, since $p^{(0)}$ is independent of *r*, we must have (59) hold for all *r* across the cross section.

We also assume an ansatz for the first-order axial fluid velocity

60 $$\begin{aligned} u_{s}^{(1)} = -\cos {\phi }\,f_{s}^{(1)}(r,s), \end{aligned}$$

for some function $f_{s}^{(1)}(r,s)>0$ yet to be determined and $-\cos {\phi }$ to ensure $u_{s}^{(1)}$ takes its minimum at ϕ=0 and maximum at $\phi = \pi$, as we expect physically. Substituting (60) into (42b) and recalling that $u_{s}^{(0)}$ is independent of *r* we obtain

61 $$\begin{aligned} \begin{aligned} 0&= -\frac{1}{r^{2}}\cos {\phi }\left( r^{2}\frac{\partial }{\partial r}\left( \left( \dot{\gamma }^{n-1}\right) ^{(0)}\frac{\partial f_{s}^{(1)}}{\partial r}\right) + r\left( \dot{\gamma }^{n-1}\right) ^{(0)}\frac{\partial f_{s}^{(1)}}{\partial r} \right. \\&\quad \left. - \left( \dot{\gamma }^{n-1}\right) ^{(0)}f_{s}^{(1)}\right) , \end{aligned} \end{aligned}$$

and, from this, acquire a second-order partial differential equation for $f_{s}^{(1)}(r,s)$

62 $$\begin{aligned} 0 = r^{2}\frac{\partial }{\partial r}\left( \left( \dot{\gamma }^{n-1}\right) ^{(0)}\frac{\partial f_{s}^{(1)}}{\partial r}\right) + r\left( \dot{\gamma }^{n-1}\right) ^{(0)}\frac{\partial f_{s}^{(1)}}{\partial r} - \left( \dot{\gamma }^{n-1}\right) ^{(0)}f_{s}^{(1)}. \end{aligned}$$

The general solution to (62) is

63 $$\begin{aligned} f_{s}^{(1)} = c_{1}(s)\,r + \frac{c_{2}(s)}{r}. \end{aligned}$$

Applying boundary condition (49f) then yields an expression for the first-order axial fluid velocity

64 $$\begin{aligned} u_{s}^{(1)} = -r\kappa ^{(0)}\cos {\phi }\,u_{s}^{(0)}, \end{aligned}$$

where $c_{2}(s) = 0$ to ensure $u_{s}^{(1)}$ is finite at r=0.

Integrating (42c) with respect to *r* and ϕ across an arbitrary cross-sectional area for $r = R^{(0)}(s)$, and applying boundary condition (49g) generates

65 $$\begin{aligned} \cos {\theta ^{(0)}} = -\frac{\text {Fr}^{2}}{\text {Re}_{\text {PL}}}\frac{1}{\left( R^{(0)}\right) ^{2}}\frac{\text {d}}{\text {d}s}\left( 3\left( R^{(0)}\right) ^{2}\left( \dot{\gamma }^{n-1}\right) ^{(0)}\frac{\text {d}u_{s}^{(0)}}{\text {d}s}\right) , \end{aligned}$$

where we have substituted in for $u_{s}^{(0)}$, $u_{r}^{(0)}$, $u_{s}^{(1)}$, and $p^{(0)}$. We remark that the 3 denotes the *Trouton ratio* for an axisymmetric filament [16]. Similarly, multiplying (43b) by $\sin {\phi }$ and (44b) by $\cos {\phi }$ and subtracting the second from the first, we obtain

66 $$\begin{aligned} \begin{aligned}&\sin {\phi }\left( \frac{1}{r^{2}}\frac{\partial }{\partial r}\left( r^{2}\sigma _{r\phi }^{(1)}\right) + \frac{1}{r}\frac{\partial \sigma _{\phi \phi }^{(1)}}{\partial \phi } + \frac{\partial \sigma _{s\phi }^{(1)}}{\partial s}\right) \\&\quad - \cos {\phi }\left( \frac{1}{r}\frac{\partial }{\partial r}\left( r\sigma _{rr}^{(1)}\right) + \frac{1}{r}\frac{\partial \sigma _{\phi r}^{(1)}}{\partial \phi } + \frac{\partial \sigma _{sr}^{(1)}}{\partial s} - \frac{\sigma _{\phi \phi }^{(1)}}{r}\right) \\&\quad - 3\,\kappa ^{(0)}\left( \dot{\gamma }^{n-1}\right) ^{(0)}\frac{\text {d}u_{s}^{(0)}}{\text {d}s} + \frac{\text {Re}_{\text {PL}}}{\text {Fr}^{2}}\sin {\theta ^{(0)}} = 0. \end{aligned} \end{aligned}$$

Integrating (66) with respect to *r* and ϕ across an arbitrary cross-sectional area for $r = R^{(0)}(s)$, and applying boundary conditions (49b) and (49d) and periodicity conditions (52a)–(52c), yields an expression for $\sin {\theta ^{(0)}}$, given by

67 $$\begin{aligned} \sin {\theta ^{(0)}} = 3\frac{\text {Fr}^{2}}{\text {Re}_{\text {PL}}}\kappa ^{(0)}\left( \dot{\gamma }^{n-1}\right) ^{(0)}\frac{\text {d}u_{s}^{(0)}}{\text {d}s}. \end{aligned}$$

Substituting (56) into (65) and (67) and recalling that $\kappa ^{(0)} = \text {d}\theta ^{(0)}/\text {d}s$ generates

68a $$\begin{aligned}&\begin{aligned} \cos {\theta ^{(0)}}&= \frac{\text {Fr}^{2}}{\text {Re}_{\text {PL}}}\frac{6}{\left( R^{(0)}\right) ^{2}}\frac{\text {d}}{\text {d}s}\left( \left( \dot{\gamma }^{n-1}\right) ^{(0)}\frac{1}{R^{(0)}}\frac{\text {d}R^{(0)}}{\text {d}s}\right) , \end{aligned} \end{aligned}$$

68b $$\begin{aligned}&\begin{aligned} \sin {\theta ^{(0)}}&= -\frac{\text {Fr}^{2}}{\text {Re}_{\text {PL}}}\frac{6}{\left( R^{(0)}\right) ^{3}}\frac{\text {d}\theta ^{(0)}}{\text {d}s}\left( \dot{\gamma }^{n-1}\right) ^{(0)}\frac{\text {d}R^{(0)}}{\text {d}s}, \end{aligned} \end{aligned}$$

where

69 $$\begin{aligned} \left( \dot{\gamma }^{n-1}\right) ^{(0)} = \left( \frac{12}{\left( R^{(0)}\right) ^{6}}\left( \frac{\text {d}R^{(0)}}{\text {d}s}\right) ^{2}\right) ^{\frac{n-1}{2}}, \end{aligned}$$

extending the power-law model to incorporate a curved centre-line. As such, we have reduced steady-state system (42a)–(52c) to a closed system of two Eqs. (68a)–(68b) satisfied by two unknowns: the leading-order filament radius and centre-line orientation, $R^{(0)}(s)$ and $\theta ^{(0)}(s)$, respectively. This system is subject to boundary conditions

70a $$\begin{aligned}&\begin{aligned}&R^{(0)}(s) = 1,\quad \qquad \qquad \text {at} \quad s=0, \end{aligned} \end{aligned}$$

70b $$\begin{aligned}&\begin{aligned}&R^{(0)}(s) = \left( \frac{\bar{u}_{\text {E}}}{U_{\text {N}}}\right) ^{\frac{1}{2}}, \quad \text {at} \quad s=1, \end{aligned} \end{aligned}$$

70c $$\begin{aligned}&\begin{aligned}&\theta ^{(0)}(s) = \frac{\pi }{2}, \quad \qquad \qquad \text {at} \quad s=1. \end{aligned} \end{aligned}$$

### Numerical scheme

We rewrite (68) as a system of first-order ordinary differential equations (ODEs) by first making a change of variables: $y_{1} = R^{(0)}$ and $y_{3} = \theta ^{(0)}$. The resulting system is given by

71a $$\begin{aligned}&\begin{aligned}&\frac{\text {d}y_{1}}{\text {d}s} = y_{2}, \end{aligned} \end{aligned}$$

71b $$\begin{aligned}&\begin{aligned}&\frac{\text {d}y_{2}}{\text {d}s} = \frac{\left( 3n-2\right) }{n}\,y_{1}\left( \frac{y_{2}}{y_{1}}\right) ^{2} + \frac{12^{\frac{1-n}{2}}\alpha _{\text {PL}}\, y_{1}^{3n}\,|y_{2}|^{1-n}}{6n}\cos {y_{3}}, \end{aligned} \end{aligned}$$

71c $$\begin{aligned}&\begin{aligned}&\frac{\text {d}y_{3}}{\text {d}s} = -\frac{12^{\frac{1-n}{2}}\alpha _{\text {PL}}\,y_{1}^{3n}\,\text {sgn}(y_{2})}{6\,|y_{2}|^{n}}\sin {y_{3}}, \end{aligned} \end{aligned}$$

where we have set

72 $$\begin{aligned} \alpha _{\text {PL}} = \frac{\text {Re}_{\text {PL}}}{\text {Fr}^{2}} = \frac{\rho L^{n+1} g}{K\bar{u}_{\text {E}}^{n}}, \end{aligned}$$

having substituted (39) in for $\text {Re}_{\text {PL}}$ and $\text {Fr}$, with $\text {sgn}(x)$ the sign function and |*x*| the modulus function. This system is subject to boundary conditions

73a $$\begin{aligned}&\begin{aligned}&y_{1}(s) = 1, \quad \qquad \qquad \text {at}\quad s=0, \end{aligned} \end{aligned}$$

73b $$\begin{aligned}&\begin{aligned}&y_{1}(s) = \left( \frac{\bar{u}_{\text {E}}}{U_{\text {N}}}\right) ^{\frac{1}{2}}, \quad \text {at} \quad s=1, \end{aligned} \end{aligned}$$

73c $$\begin{aligned}&\begin{aligned}&y_{3}(s) = \frac{\pi }{2}, \quad \quad \quad \quad \,\,\text {at} \quad s=1. \end{aligned} \end{aligned}$$

There remains three unknown parameters in the system, a leading-order dimensionless group, $\alpha _{\text {PL}}$, given by (72), a velocity ratio, $\bar{u}_{\text {E}}/U_{\text {N}}$, imposed at the boundary, given by (73b), and flow behaviour index, *n*. Given that $\alpha _{\text {PL}}$ appears at leading order, we treat it as an ${\mathcal {O}}(1)$ quantity and, for definiteness, adopt a representative range of 0.2–5 [10]. Paxton et al. [10] report that extrusion typically operates with $\bar{u}_{\text {E}}/U_{\text {N}}$ close to 1; accordingly, we restrict our attention to the steady regime $\bar{u}_{\text {E}}\lesssim U_{\text {N}}$ and take $\bar{u}_{\text {E}}/U_{\text {N}}\in (0,1)$. Finally, treating Nivea Crème as a highly printable material, we adopt the measured value n=0.552 reported by Paxton et al. [10], corresponding to a moderately shear-thinning material, as defined in Sect. 1. Boundary-value problem (71)–(73) was then solved via implementation of the versatile ODE solver in $\textsc {Matlab}^{\circledR }$, ode45. A shooting method was applied via fzero, a scalar root-finding algorithm in $\textsc {Matlab}^{\circledR }$. Unknown condition, $y_{2}(s=1) = \beta$, was iteratively adjusted until boundary condition, $y_{1}(s=0) = 1$, was satisfied. This was executed on an $\hbox {Intel}^{\circledR }$ Core^™^ i7-11800 H CPU (16 GB RAM), with each shooting computation requiring less than a second. Upon obtaining the solution, (46), given by

74 $$\begin{aligned} \int _0^{1} \cos {y_{3}}(s)\,\text {d}s = \frac{H}{L}, \end{aligned}$$

was used to obtain *H*/*L*.

### Experimental methods

Like many hydrogels, Nivea Crème is predominantly water-based and can therefore be modelled as an incompressible fluid, making it a convenient benchmark material for an initial assessment [24].

Nivea Crème was printed via an INKREDIBLE+ 3D bioprinter (CellInk, Gothenburg, Sweden) with a 20G conical nozzle made of polypropylene (inner diameter 4.02 mm, outer diameter 0.58 mm, length 32 mm [25]), 3 mm nozzle offset, and a 6 mm/s nozzle travel speed, onto a build platform held at room temperature ($22\pm {1}^{\circ }$C). To approximate changes in extrusion speed, $\bar{u}_{\text {E}}$, the applied pressure was varied, exploiting the direct proportionality observed by Tansell et al. [19]. As such, six different air pressures were considered (55,56,…,60 kPa) with six lines printed in total. For each applied pressure, the line print was repeated three times and an average width taken. In each case, the measurement was taken from the middle of the printed line. Table 1 summarises the process printing parameters and rheological values of Nivea Crème used.

**Table 1:** Material and printing process parameters used in experiments

Material	Nivea Crème
Material Density, ${\rho }$ (kg/$\text {m}^{3}$)	972 [23]
Inner Nozzle Radius, ${r_{\text {i}}}$ (mm)	2.01 [25]
Outer Nozzle Radius, ${r_{\text {o}}}$ (mm)	0.29 (20G)
Nozzle Length, ${L_{\text {c}}}$ (mm)	32
Nozzle Travel Speed, ${U_{\text {N}}}$ (mm/s)	6
Nozzle Offset, *H* (mm)	3
Applied Air Pressure, ${\Delta P}$ (kPa)	55, 56, 57, 58, 59, 60
Syringe Temperature (C)	22
Build Platform Temperature (C)	22

As derived by Tansell et al. [19] for a conical nozzle and non-Newtonian fluid, the average extrusion speed, $\bar{u}_{\text {E}}$, is given by

75 $$\begin{aligned} \bar{u}_{\text {E}} = \frac{r_{\text {i}}^{3}r_{\text {o}}}{4}\left( \frac{4n}{3n+1}\right) \left[ \frac{3n\,(r_{\text {i}}-r_{\text {o}})\Delta P}{2KL_{\text {c}}\left( r_{\text {i}}^{3n} - r_{\text {o}}^{3n}\right) }\right] ^{\frac{1}{n}}, \end{aligned}$$

where $r_{\text {i}}$, $r_{\text {o}}$, $L_{\text {c}}$ denote the inner radius, outer radius, and length of the conical nozzle and ΔP the applied air pressure. To avoid selecting a value for consistency index, *K*, (75) is raised to the power *n* and multiplied by *K* before substitution into (72). This yields

76 $$\begin{aligned} \alpha _{\text {PL}} = \frac{2^{2n+1}}{3n}\left( \frac{3n+1}{4n}\right) ^{n}\left( \frac{r_{\text {i}}^{3n} -r_{\text {o}}^{3n}}{r_{\text {i}}^{3n}r_{\text {o}}^{n}}\right) \left( \frac{L}{H}\right) ^{n+1}\frac{\rho H^{n+1}L_{\text {c}}\,g}{(r_{\text {i}}-r_{\text {o}})\Delta P}. \end{aligned}$$

For each experiment, the corresponding process parameters were substituted into (76) to approximate a value of $\alpha _{\text {PL}}$.

Each experiment was captured using a Dino-Lite Digital Microscope (AnMo Electronics Corporation, Taiwan), and analysed in ImageJ (NIH, MD, USA). All subsequent statistical analyses were performed in $\textsc {Matlab}^{\circledR }$ (MathWorks, MA, USA) version 23.2.0.2380103 (R2023b). Regression analysis was conducted using the Curve Fitting Toolbox^TM^.

## Results and discussion

**Fig. 3:**
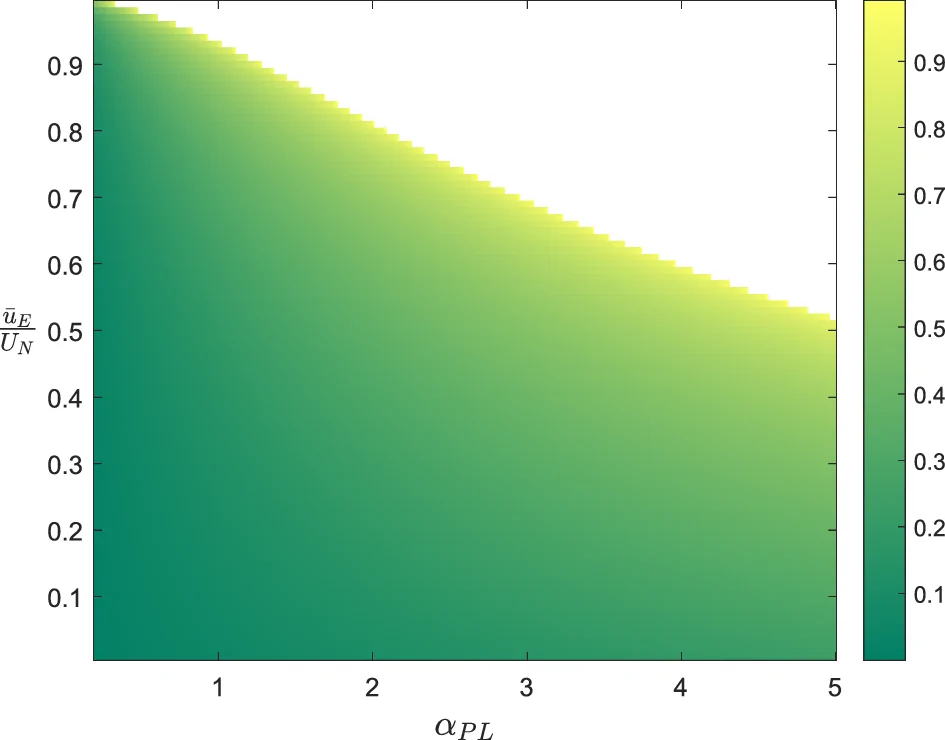
Heatmap presenting *H*/*L* as the velocity ratio, $\bar{u}_{\text {E}}/U_{\text {N}}$, and $\alpha _{\text {PL}}$ are varied from 0.01 to 0.99 and 0.2 to 5, respectively. White denotes where a solution could not be provided

**Fig. 4:**
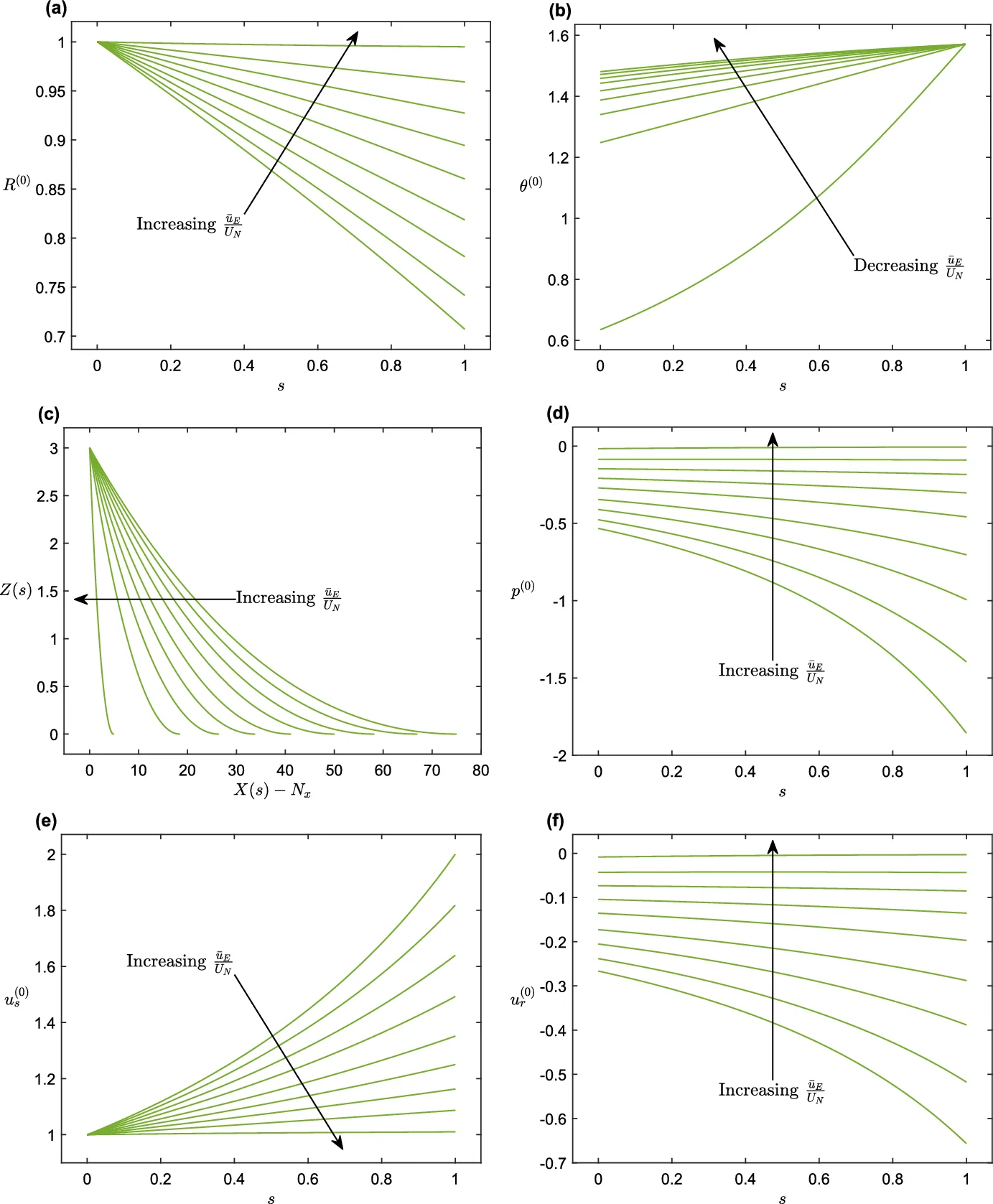
Variation along arc length *s* of **a** $R^{(0)}$, **b** $\theta ^{(0)}$, **c** geometry of the centre-line (dimensionalised with H=3 mm), **d** $p^{(0)}$, **e** $u_{s}^{(0)}$, and **f** $u_{r}^{(0)}$, for $\alpha _{\text {PL}} = 0.2$ and $u_{\text {E}}/U_{\text {N}}$ varied from 0.5 to 0.99

**Fig. 5:**
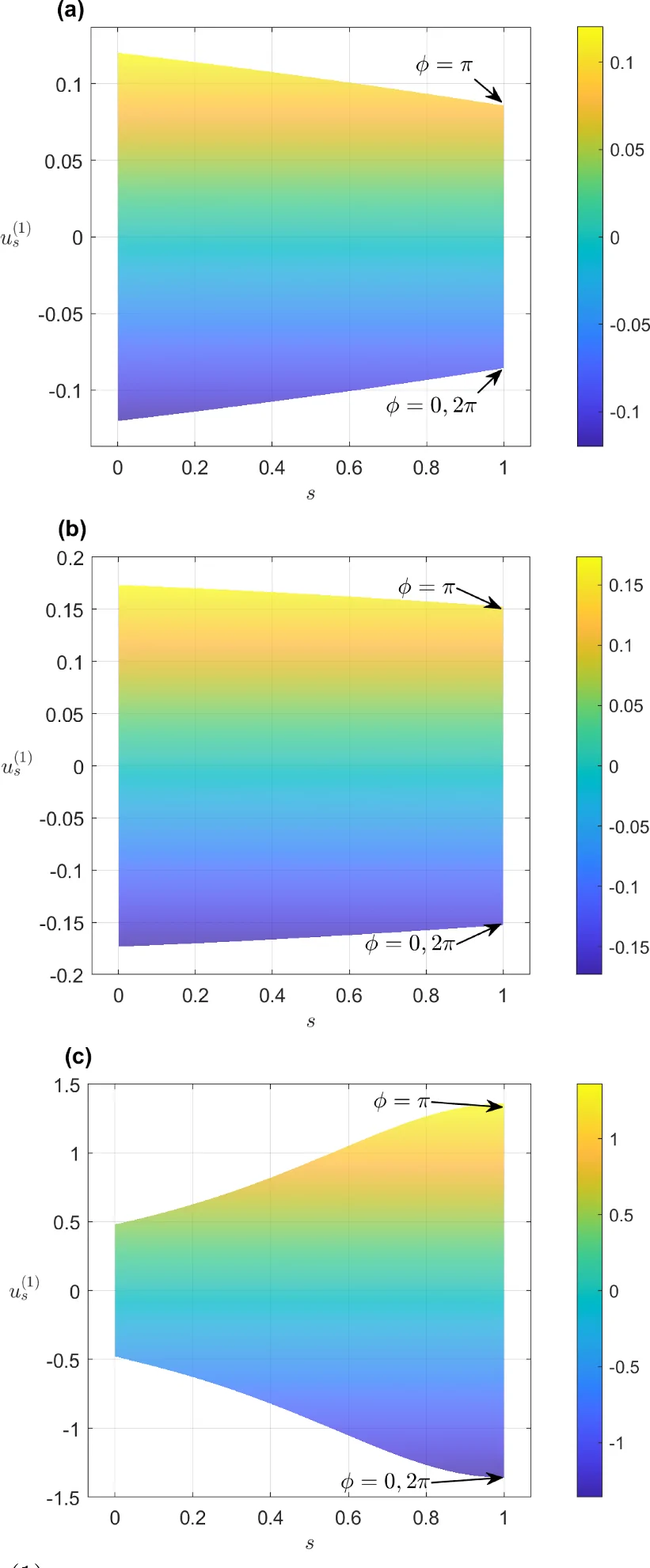
Variation of $u_{s}^{(1)}$ along arc length *s* as ϕ increases within filament cross section, for $\alpha _{\text {PL}} = 0.2$ and $\bar{u}_{\text {E}}/U_{\text {N}}$ equal to **a** 0.5, **b** 0.74, and **c** 0.99

Figure 3 presents dimensionless results for *H*/*L* as the velocity ratio, $\bar{u}_{\text {E}}/U_{\text {N}}$, and $\alpha _{\text {PL}}$ are varied from 0.01 to 0.99 and 0.2 to 5, respectively. The white region of parameter space indicates where $\theta ^{(0)}(s<1)$ is very close to 0. Since $\theta ^{(0)}(s)$ must divert to π/2 at s=1 [as imposed by boundary condition (73c)], bending stresses within the boundary layer along the surface of the build platform become of greater influence to the dynamics and, therefore, a steady catenary-like solution could not be provided. Furthermore, due to boundary condition (73b), each value of $\bar{u}_{\text {E}}/U_{\text {N}}$ corresponds to a radius, $R^{(0)}(s)$, evaluated at s=1.

Selecting a specific combination of $\bar{u}_{\text {E}}/U_{\text {N}}$ and $\alpha _{\text {PL}}$, we can determine unknown variables in the system. Figures 4 and 5 exhibit results for $\alpha _{\text {PL}}$ equal to 0.2 as $\bar{u}_{\text {E}}/U_{\text {N}}$ is varied from 0.5 to 0.99. For clarity, Fig. 4c is dimensionalised by assuming *H* to be 3 mm, the nozzle offset used in the experiments (see Table 1). Figures  4a, 4b, 4d, 4e, and 4f are presented in dimensionless form.

### Model results

Variation of $R^{(0)}$(s) along the arc length *s* can be found in Fig. 4a, for varying velocity ratio, $\bar{u}_{\text {E}}/U_{\text {N}}$. For a fixed nozzle travel speed, $U_{\text {N}}$, a reduction in $\bar{u}_{\text {E}}$ increases the deviation between the printed filament radius on the build platform (at s=1) and the radius imposed at the nozzle tip (at s=0). When material is supplied more slowly than the nozzle advances, the filament experiences greater extensional deformation during deposition. Conservation of mass then requires a reduction in cross-sectional area, producing a smaller printed filament radius. Since we require the printed filament radius to be as close to that imposed at the nozzle tip as possible (termed optimal resolution in this paper), an extrusion speed close to the nozzle travel speed is necessary (as highlighted by Paxton et al. [10]).

In an experimental set-up, the extrusion speed is rarely known a priori and can be a difficult variable to measure [10]. Tansell et al. [19] deduced the extrusion speed to be dependent on applied pressure, nozzle geometry, and flow behaviour and consistency indices, *n* and *K*. This shortlist of printing parameters agreed with those obtained experimentally (e.g. He et al. [26], Schwab et al. [27], and Suntornnond et al. [28]). As such, for a fixed nozzle geometry and material, (75) indicates that the applied pressure, ΔP (a parameter required by a bioprinter), should be adjusted to fine-tune the extrusion speed. Returning to Fig. 4a, an increase in ΔP would result in an increase in $\bar{u}_{\text {E}}$, as desired.

If the extrusion speed was chosen to be close to the nozzle travel speed, the filament centre-line orientation adjusts accordingly, and the angle between its tangent and the downward vertical tends towards zero. As the extrusion speed approaches the nozzle travel speed, the filament experiences less longitudinal stretching and therefore requires less horizontal displacement to accommodate nozzle motion. Gravity consequently becomes the dominant influence on filament shape, causing the centre-line to approach a vertical configuration. In this limit, the filament becomes nearly vertical (see Fig. 4b). Figure 4c captures this centre-line behaviour as the velocity ratio, $\bar{u}_{\text {E}}/U_{\text {N}}$, is varied. In addition, Fig. 4f reveals the radial fluid velocity, $u_{r}^{(0)}$, to approach zero as $\bar{u}_{\text {E}}/U_{\text {N}}$ is increased; the filament gradually stops shrinking. The reduction in radial velocity reflects a decrease in filament thinning. As the extrusion and nozzle travel speeds become comparable, less extensional deformation is required and the filament radius approaches a steady value closer to that imposed at the nozzle tip. As such, to maintain a steady fluid flow and maximise print resolution and shape fidelity, we recommend an average extrusion speed, $\bar{u}_{\text {E}}$, approximately equal to the nozzle moving speed, $U_{\text {N}}$. To achieve this, the air pressure applied to the material should be adjusted accordingly.

**Table 2:** Average measurements for filament radius (row 1), in millimetres (mm), filament length (row 2), in millimetres (mm), ratio between nozzle offset and filament length (row 3), and value of $\alpha _{\text {PL}}$ (row 4), for Nivea Crème using a 20G (0.58 mm) conical nozzle, 6 mm/s nozzle travel speed, and a 3 mm nozzle offset

	Applied Air Pressure (kPa)					
	55	56	57	58	59	60
${R_{\textrm{L}}}$	$0.244 \pm {0.005}$	$0.260 \pm {0}$	$0.269 \pm {0}$	$0.279 \pm {0}$	$0.285 \pm {0.005}$	$0.289 \pm {0}$
*L*	$35.5 \pm {2.8}$	$31.7 \pm {1.3}$	$29.9 \pm {1.5}$	$25.1 \pm {1.8}$	$15.5 \pm {1.6}$	$5.80 \pm {0.41}$
*H*/*L*	$0.0848 \pm {0.007}$	$0.0946 \pm {0.004}$	$0.100 \pm {0.005}$	$0.120 \pm {0.009}$	$0.196 \pm {0.020}$	$0.519 \pm {0.038}$
${\alpha _{\text {PL}}}$	$4.50 \pm {0.55}$	$3.70 \pm {0.23}$	$3.32 \pm {0.25}$	$2.48 \pm {0.28}$	$1.15 \pm {0.19}$	$0.247 \pm {0.027}$

### Preliminary experimental validation

**Fig. 6:**
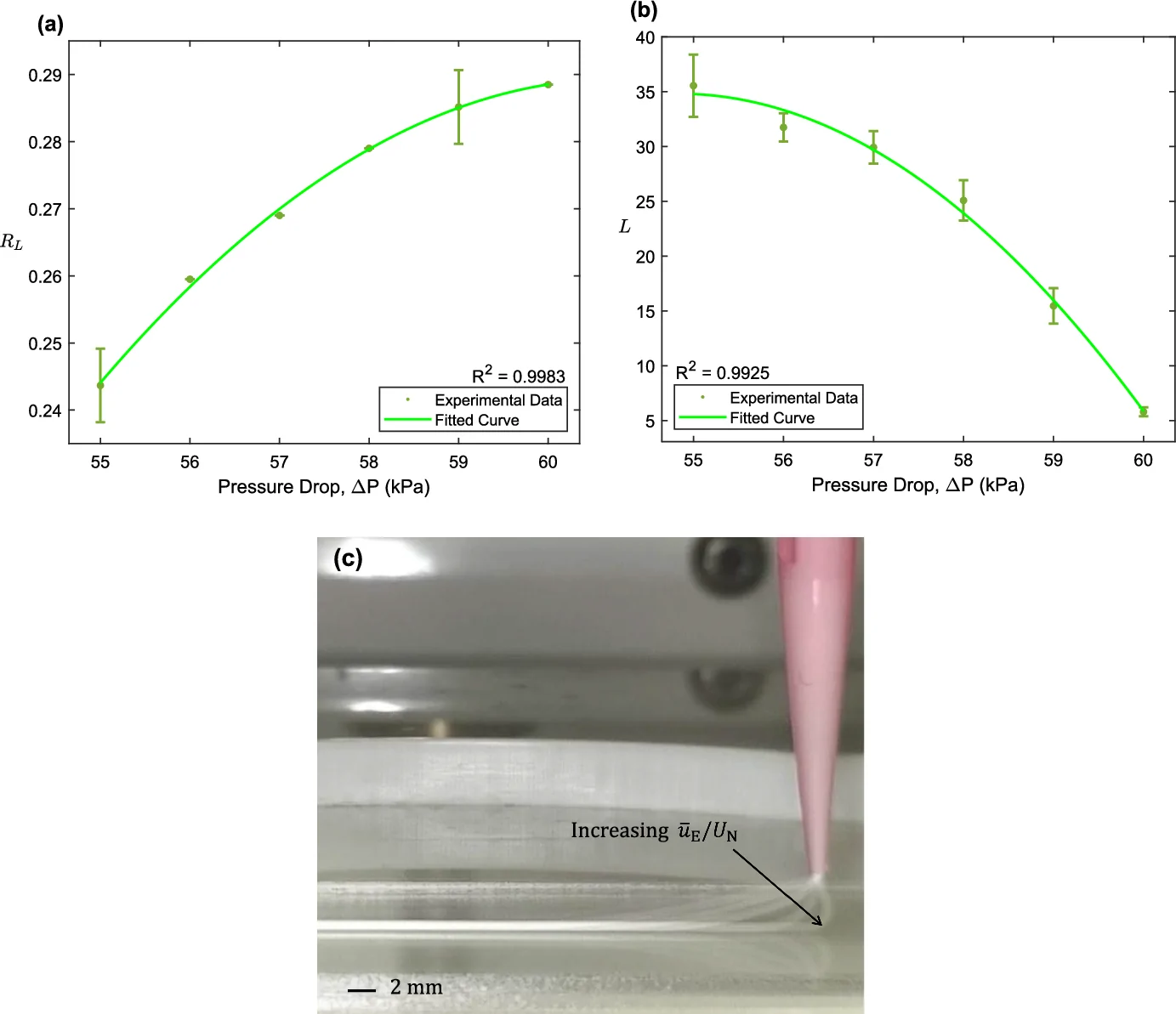
Regression analysis determining relationship between applied air pressure, ΔP (kPa), and filament **a** radius, $R_{L}$ (mm), and **b** length, *L* (mm), of Nivea Crème, with a 20G conical nozzle, 3 mm nozzle offset, and a 6 mm/s nozzle travel speed. Curve fitting applied with form $y = ax^{2} + bx + c$, with *a*, *b*,  and *c* constant coefficients given in Table S1 in Supplementary Information. **c** Filament centre-line orientation for increasing $\bar{u}_{\text {E}}/U_{\text {N}}$

As a preliminary validation of the model, we conducted a series of experiments to compare theoretical predictions with observed results. The theoretical model accentuates the slenderness of the filament and assumes a small $\varepsilon ^{n}$, specifically, $\varepsilon ^{n} = {\mathcal {O}}(10^{-2})$, a scale uncommon in 3D printing (where, typically, $H\sim (2/3)D_{\text {N}}$ [10]). To print at this scale, the model recommends use of a velocity ratio, $\bar{u}_{\text {E}}/U_{\text {N}}$, closer to 1. Doing so, a filament with a radius closer to the nozzle tip radius, $R_{\text {N}}$, and a filament centre-line orientation closer to vertical (i.e. *L* approaches *H*) will be produced. Fixing nozzle geometry ($r_{\text {i}}$, $r_{\text {o}}$, and $L_{\text {c}}$), nozzle offset, *H*, and nozzle travel speed, $U_{\text {N}}$, we varied the air pressure, ΔP, applied to the hydrogel to generate data for preliminary model validation purposes. Table 2 presents the average measurements taken in the experiments for the filament radius, $R_{L}$, and length, *L*, of Nivea Crème. Experimental values of *H*/*L* and $\alpha _{\text {PL}}$ are also presented. Figures 6a and 6b presents the results of a regression analysis, where a quadratic polynomial is fitted (see Table S1 in Supplementary Information), with Fig. 6c an image overlay of the filament centre-line orientation as $\bar{u}_{\text {E}}/U_{\text {N}}$ is increased.

#### Reinterpretation of experimental observation

The model captures experimental observation and provides a methodology through which we can extend understanding of how process parameters influence the complex rheology of a material.

Experimentally, when $\bar{u}_{\text {E}}<U_{\text {N}}$, we observe filaments of material adhering to the build platform (provided the pressure is sufficient enough) and stretching to produce printed radii below $r_{\text {o}}$, due to conservation of mass. As the applied pressure is increased (or $\bar{u}_{\text {E}}\rightarrow U_{\text {N}}$), an optimal trade-off between the air pressure (or extrusion speed) and nozzle travel speed is located and printed radii closer to $r_{\text {o}}$ obtained as a result. In this way, catenary-like filaments are formed. This behaviour is captured and predicted in Figs. 6c and 4c, respectively. Figure 6a reveals the printed filament radius converging towards the optimal radius, the nozzle tip radius, $r_{\text {o}}$, as the applied pressure, ΔP, is increased (or $\bar{u}_{\text {E}}\rightarrow U_{\text {N}}$), as predicted in Fig. 4a. Whilst, Figs. 6b and 6c presents confirmation of that obtained theoretically in Figs. 4b and 4c, i.e. as ΔP is increased, the orientation of the filament centre-line approaches the vertical position.

With model results presenting good agreement with experiment, we hypothesise that the methodology has potential to recommend air pressures likely to generate printed radii close to the nozzle tip radius (having specified a material, nozzle geometry, and a nozzle travel speed).

### Applications of modelling framework

Choosing an appropriate value of $\alpha _{\text {PL}}$ (as approximated in the experiments) and using Fig. 3 to determine the corresponding $\bar{u}_{\text {E}}/U_{\text {N}}$, we performed a preliminary validation of the model as tool for prediction. Given $\bar{u}_{\text {E}}/U_{\text {N}}$, the filament radius, $R^{(0)}(s = L)$, was approximated via use of the dimensionalised boundary condition

77 $$\begin{aligned} R^{(0)}(s) = R_{\text {N}}\left( \frac{\bar{u}_{\text {E}}}{U_{\text {N}}}\right) ^{\frac{1}{2}}, \quad \text {at} \quad s=L, \end{aligned}$$

and compared with its experimental value, $R_{L}$. With the overarching aim of defining process parameter combinations that deliver optimal resolution for a given material and set-up—i.e. the bioink’s window of printability—we restricted our initial assessment to results obtained at 60 kPa, the pressure corresponding to the experimental optimum ($R_{L}\approx r_{\text {o}}$). Since $\alpha _{\text {PL}}$ falls within the range 0.2–5 in this case (see Table 2), we confirm that the model is applicable. Table 3 presents the results of this analysis.

With a percentage error of 0.32%, the model appears to provide a good prediction for the filament radius, $R^{(0)}(s)$, on the build platform (at s=L), while indicating that Fig. 3 has potential to recommend a window of printability for a specific material and set-up.

Unlike the flux-based framework developed in Tansell et al. [19], which relates applied pressure to extrusion speed and predicts printed dimensions through conservation of mass alone, the present model explicitly captures the evolution of the filament between the nozzle tip and build platform. Consequently, the model provides additional information regarding filament geometry, centre-line orientation, pressure variation, and velocity fields throughout the extrusion process (see Figs. 4 and 5). This enables theoretical identification of windows of printability based on filament dynamics, rather than solely on nozzle-exit conditions, providing a more physically representative basis for predicting print resolution.

**Fig. 7:**
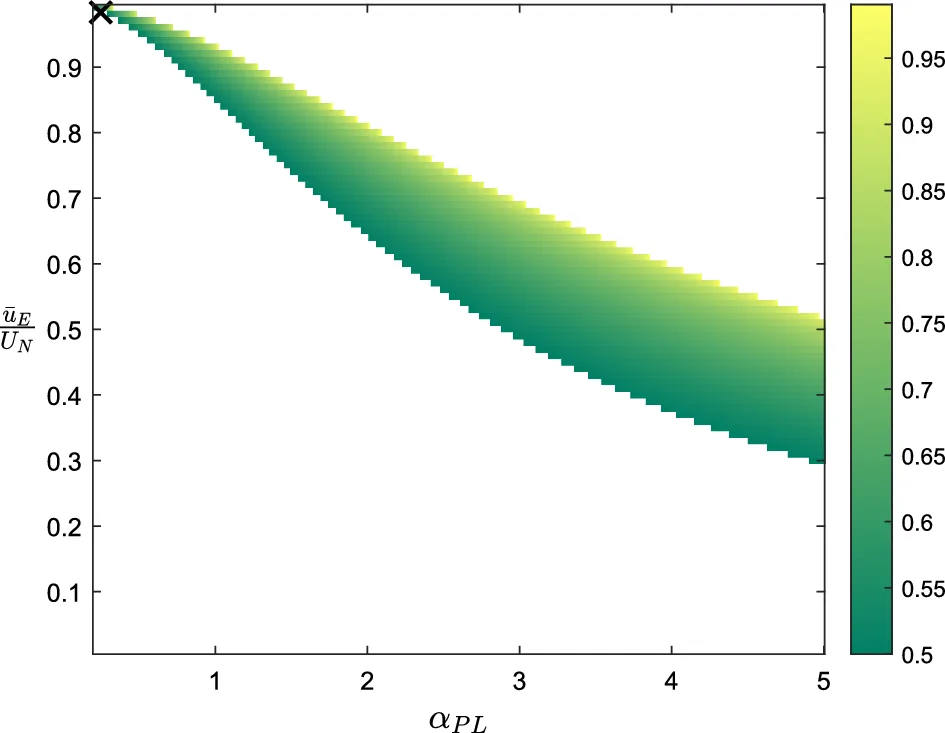
Heatmap restricting *H*/*L* to between 0.5 and 1 as the velocity ratio, $\bar{u}_{\text {E}}/U_{\text {N}}$, and $\alpha _{\text {PL}}$ are varied from 0.01 to 0.99 and 0.2 to 5, respectively. Black cross represents $(\alpha _{\text {PL}}, \bar{u}_{\text {E}}/U_{\text {N}})$ corresponding to the combination of process parameters providing optimal resolution in Nivea Crème experiments

Nivea Crème was chosen as a suitable material for a preliminary model validation due to its highly printable properties. As such, we chose to restrict parameter space by a lower bound corresponding to its optimal experimental *H*/*L*, approximated to be 0.519. Expecting *H*/*L* to approach 1 as the optimal combination is reached (see Fig. 4c), an upper bound of 1 was adopted. Using 0.5 as the lower bound and 1 as the upper bound, the non-Newtonian model produced the window of printability shown in Fig. 7. Values below this range correspond to excessive filament stretching and thinning, leading to poor shape fidelity and printed radii that deviate significantly from the nozzle tip radius. The upper white region remains the same as that identified in Fig. 3, corresponding to parameter combinations for which a steady catenary-like solution could not be obtained. Consequently, the highlighted region represents operating conditions that maintain both stable filament deposition and printed radii close to those imposed by the nozzle geometry.

**Table 3:** Straight line print using Nivea Crème with a 20G conical nozzle, 6 mm/s nozzle travel speed, 3 mm nozzle offset, and a 60 kPa air pressure

	$${R^{(0)}(s=L)}$$
Experimental Results (mm)	$0.289 \pm {0}$
Model Predictions (mm)	$0.286 \pm {0.001}$
Percentage Error (%)	0.323

Row 1 presents measurements for filament radius, $R^{(0)}(s=L)$, where $\alpha _{\text {PL}}$ was approximated as 0.247. Row 2 presents model predictions for this $\alpha _{\text {PL}}$, with row 3, the percentage error between experimental and model results

**Fig. 8:**
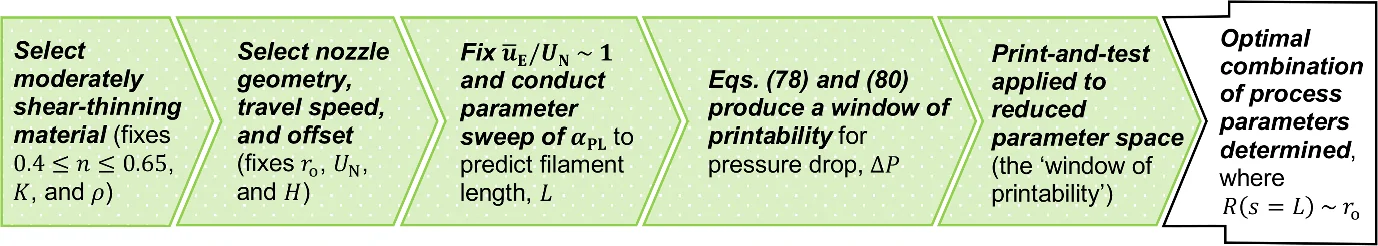
Workflow implementing the model to establish a ‘*window of printability*’ for applied pressure, ΔP

If an engineer were to choose a moderately shear-thinning material ($0.40\le n \le 0.65$, *K* and ρ), nozzle geometry (e.g. $r_{\text {i}}$, $r_{\text {o}}$, and $L_{\text {c}}$, for a conical nozzle), nozzle travel speed, $U_{\text {N}}$, and nozzle offset, *H*, the model could be employed to determine sets of extrusion speeds, $\bar{u}_{\text {E}}$, likely to generate optimal resolutions. Figure 8 presents a flowchart of how the user may apply the model in this way.

Since extrusion speed is rarely known in advance during experiments, empirical expressions derived in previous work (see Tansell et al. [19]) are used to link air pressure and extrusion speed. This provides a practical way to connect the model predictions with experimental conditions. With required input provided by engineers, (76) reduces to

78 $$\begin{aligned} \Delta P = \frac{L^{n+1}}{\alpha _{\text {PL}}\,A_{\alpha _{\text {PL}}}}, \end{aligned}$$

with

79 $$\begin{aligned} A_{\alpha _{\text {PL}}} = \frac{3n}{2^{2n+1}}\left( \frac{4n}{3n+1}\right) ^{n}\left( \frac{r_{\text {i}}^{3n}r_{\text {o}}^{n}}{r_{\text {i}}^{3n} - r_{\text {o}}^{3n}}\right) \frac{r_{\text {i}}-r_{\text {o}}}{\rho \,L_{\text {c}}\,g}, \end{aligned}$$

fixed. While (75) provides

80 $$\begin{aligned} \Delta P = \frac{A_{\bar{u}_{\text {E}}}}{\bar{u}_{\text {E}}^{n}}, \end{aligned}$$

for a conical nozzle type (i.e. $r_\text {i}>r_{\text {o}}$), with

81 $$\begin{aligned} A_{\bar{u}_{\text {E}}} = \frac{2^{2n+1}}{3n}\left( \frac{3n+1}{4n}\right) ^{n}\left( \frac{r_{\text {i}}^{3n} - r_{\text {o}}^{3n}}{r_{\text {i}}^{3n}r_{\text {o}}^{n}}\right) \frac{KL_{\text {c}}}{r_{\text {i}}-r_{\text {o}}}, \end{aligned}$$

fixed. We note that other axisymmetric nozzle types can be readily implemented via use of Eq. (2.5) in Tansell et al. [19], if required. To optimise the resolution, we require $\bar{u}_{\text {E}}/U_{\text {N}}$ close to 1. Fixing $\bar{u}_{\text {E}}/U_{\text {N}}\sim 1$ and varying $\alpha _{\text {PL}}$ from 0.2 to 5, the model predicts filament length, *L*, for each combination, and Eqs. (78) and (80) provide corresponding air pressures which must then match to some small tolerance. Doing so identifies a window of printability for the applied pressure, providing engineers with a reduced set of process parameter combinations to explore via print-and-test. Returning to Fig. 7, windows of printability produced via this methodology (in the case n=0.552) then correspond to ‘*lines of optimality*’ within the generic heatmap for particular materials and set-ups.

Paxton et al. [10] also generate windows of printability, but focus on predicting optimal extrusion speeds having fixed the material and varied straight nozzle size and air pressure. However, with stronger emphasis on experimental observation, their methodology is limited to a particular material and set-up. For example, their derived empirical expression is restricted to a straight nozzle geometry, which deviates from the ultimate goal of optimising printability for cell-laden materials [13, 29]. Based on the incompressible Navier–Stokes equations, our model generalises their methodology and arrives at windows for arbitrary moderately shear-thinning materials ($0.40\le n \le 0.65$). As such, we vary $\alpha _{\text {PL}}$ (dependent on material properties and nozzle geometry) and a velocity ratio, $\bar{u}_{\text {E}}/U_{\text {N}}$ (directly proportional to applied pressure), and instead focus on the nozzle offset to filament length ratio, *H*/*L*, which then corresponds to air pressure [via (78) and (80)]. In doing so, we enhance the applicability of the methodology by making it transferable across material and experimental set-up. In fact, a strong resemblance in shape and position is observed between Fig. 7 and that was generated by Paxton et al. [10] for Nivea Crème, providing further support for our recommendation.

#### Experimental implications

With promise as a tool for prediction, we state experimental implications of the model for the pneumatic extrusion-based bioprinting process here.

Owing to its ability to predict generalised windows of printability directly from material properties, nozzle geometry, travel speed, and offset, the model provides a route to identifying extrusion speeds likely to yield optimal resolution without extensive print-and-test iterations. Through the empirical expression linking nozzle geometry and applied pressure to extrusion speed [19], these theoretical predictions map readily onto process parameters used in pneumatic extrusion-based bioprinters, enabling experimentalists to translate model output directly into printer settings. Because the framework is grounded in theory rather than experimental observation, it remains transferable across bioinks and set-ups, supporting reproducibility and facilitating collaboration between laboratories and researchers. Moreover, by quantifying the flow and shear environments associated with different printing regimes, the model offers a solid framework for interrogating how printability and resolution coexist with cell viability constraints, and for identifying conditions in which these aims may diverge. Its formulation also accommodates integration with complementary models—such as those describing filament spreading, swelling, or flow instabilities during extrusion—allowing future work to pinpoint stages of the printing process where parameter adjustment may be required. Finally, since $\alpha _{\text {PL}}$ incorporates ρ, *n*, and *K*, the same framework could potentially be used to infer rheological profiles that favour desirable printing outcomes, offering a theoretical pathway towards the model-driven design of future bioinks.

### Limitations and future work

The power-law model was used to represent apparent viscosity. Although empirical and limited in scope, it is routinely fitted to rheometric data and is therefore appropriate for modelling the shear-thinning behaviour of many bioinks [10, 19]. However, it does not capture yield stress behaviour, and therefore may predict flow at low shear stresses where viscoplastic materials would instead behaviour elastically. This limitation is particularly relevant for bioinks that exhibit a finite yield stress, such as those described by Herschel–Bulkley-type constitutive laws.

The present framework focuses on regimes in which shear rates are sufficiently high for shear-thinning behaviour to dominate, such that a power-law approximation remains appropriate [10]. Nevertheless, the governing equations are formulated in a general manner and could be extended to incorporate alternative constitutive laws, such as Herschel–Bulkley, to account for yield stress behaviour [30]. Such an extension would enable modelling of additional phenomena, including delayed flow onset and improved prediction of filament stability and shape fidelity, although it would introduce additional parameters and complexity into the analysis.

**Fig. 9:**
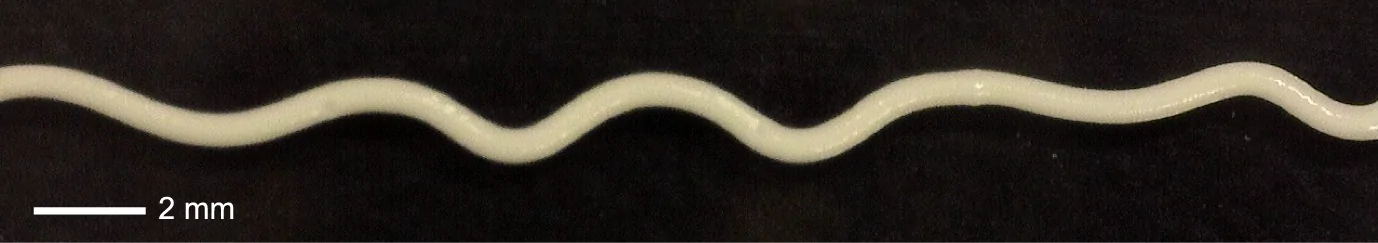
Straight line print of Nivea Crème with a 20G conical nozzle, 6 mm/s nozzle travel speed, and a 3 mm nozzle offset, revealing unstable behaviour arising when extrusion is faster than nozzle travel speed

For the asymptotic framework to hold, the flow behaviour index must satisfy $\varepsilon< \varepsilon ^{n} < 1$, making moderately shear-thinning materials (i.e. $0.40\le n \le 0.65$) suitable. Figure 3 uses n=0.552, reported for Nivea Crème by Paxton et al. [10], though similar values of *n* within this range could equally be explored.

Model validation was limited to a single material and experimental set-up, and although the model achieved a small prediction error (around 0.32%), broader testing across materials and process parameter ranges is needed. Future work will expand the dataset and include sensitivity analysis to quantify parameter influence and assess robustness.

Experimentally, as the extrusion speed is reduced relative to the nozzle travel speed, the filament undergoes excessive extensional stretching as the nozzle advances faster than material is supplied. This results in pronounced filament thinning and reduced shape fidelity, causing the printed filament radius to deviate significantly from that imposed by the nozzle geometry. Simultaneously, the filament length increases relative to the nozzle offset, such that L≫H and therefore H/L≪1. These conditions lie outside the window of printability identified in Fig. 7, defining a stretching-dominated regime associated with poor print quality.

By contrast, unstable behaviour—such as coiling and meandering—was observed experimentally when the extrusion speed approached or exceeded the nozzle travel speed (Fig. 9). In this regime, bending stresses and boundary layer structure near the build platform become dynamically important, as described in viscous thread analyses [21]. The present work focuses instead on the intermediate, axial-tension-dominated regime, applicable when $\bar{u}_{\text {E}}\lesssim U_{\text {N}}$, where bending resistance is negligible and catenary-type profiles arise. Similar bending-resistance-free slender jet solutions have been reported by Chiu-Webster and Lister [31], as well as in the contexts of rotary spinning [17, 32] and fibre pulling [33–35]. As $\bar{u}_{\text {E}}\rightarrow U_{\text {N}}$, bending stresses become essential and a boundary-layer-type singular perturbation emerges. Extending the model to include bending moments and inner–outer matching would allow exploration of regimes where $\bar{u}_{\text {E}}>U_{\text {N}}$ or $\bar{u}_{\text {E}}\sim U_{\text {N}}$, capturing the onset of the unstable behaviour observed experimentally. The predicted window of printability therefore lies between these two limits: a stretching-dominated regime at low velocity ratios and an instability-dominated regime at high velocity ratios.

## Conclusion

Considering a power-law fluid, time-dependent and steady-state models for a slender filament under pneumatic extrusion were derived using an arc length coordinate system. Asymptotic reduction of the incompressible Navier–Stokes equations yielded a closed system describing the filament radius and centre-line orientation. The model contains three unknown parameters: a generalised dimensionless group, $\alpha _{\text {PL}}$ (a ratio of $\text {Re}_{\text {PL}}/\text {Fr}^{2}$); a velocity ratio, $\bar{u}_{\text {E}}/U_{\text {N}}$; and flow behaviour index, *n*. Fixing *n* and varying $\alpha _{\text {PL}}$ and $\bar{u}_{\text {E}}/U_{\text {N}}$, we generated a heatmap of the nozzle offset to filament length ratio, *H*/*L*, enabling reinterpretation of experiments and prediction of the extruded filament radius, $R^{(0)}(s=L)$.

The model is valid for moderately shear-thinning materials (i.e. $0.40\le n \le 0.65$), consistent with the required asymptotic ordering $\varepsilon< \varepsilon ^{n} < 1$. For preliminary validation, Nivea Crème was extruded across a range of applied pressures; it was selected because it is widely regarded as a highly printable, stable demonstration ink with consistent composition across temperatures. The model showed encouraging agreement with measured filament radii, although broader datasets and accompanying sensitivity analyses are required for a more comprehensive assessment.

A window of printability was constructed to guide engineers towards parameter combinations that maximise resolution while reducing reliance on print-and-test methodologies. The predicted window is bounded by two distinct physical mechanisms: excessive extensional stretching and filament thinning at low velocity ratios, and instability-driven behaviour, such as coiling and meandering, as the extrusion speed approaches or exceeds the nozzle travel speed. Consequently, the optimal operating region lies between these limits, where deposition remains stable and the printed filament radius remains close to that imposed by the nozzle tip. This framework generalises the empirical windows of Paxton et al. [10], while also identifying a region of parameter space incompatible with the current model, suggesting that future extensions should incorporate bending stresses near the build platform.

Overall, by reducing dependence on empirical estimation and highlighting the value of theoretical modelling, this unique framework offers a practical tool to improve the efficiency and design flexibility of extrusion-based bioprinting.

## Supplementary Information

Below is the link to the electronic supplementary material.

**Supplementary information:** A supplementary information file to support model derivation and validation is provided alongside the main article (pdf 239KB)

## Data Availability

All data supporting the findings of this study are included within the article (and any Supplementary Information). Raw experimental data, MATLAB scripts were used to solve the model and validate, and an accessible software library with accompanying graphical user interface (implemented in Python), DOI: 10.25500/edata.bham.00001614.
