# Supplementary material for: An asymptotic slender-body model for predicting print resolution in extrusion-based bioprinting
Source: J Eng Math. 2026 Aug 12;159(1):15. doi: 10.1007/s10665-026-10544-0 (PMC13468979; doi:10.1007/s10665-026-10544-0)
Supplement: Supplementary file 1 [file 10665_2026_10544_MOESM1_ESM.pdf]

An asymptotic slender-body model for predicting print resolution in extrusion-based bioprinting

Supplementary Information

Amy V Tansell*[1,2], Galane J Luo[1], Paraskevi Diakourti[1,3], Nasim Mahmoodi[4], Lauren EJ Thomas-Seale[3], and Rosemary J Dyson[1]

[1] *School of Mathematics, University of Birmingham, Birmingham B15 2TT, UK*
[2] *Centre for Doctoral Training in Topological Design, University of Birmingham, Birmingham B15 2TT, UK*
[3] *Department of Mechanical Engineering, School of Engineering, University of Birmingham, Birmingham B15 2TT, UK*
[3] *Department of Electronic Electrical and Systems Engineering, School of Engineering, University of Birmingham, Birmingham B15 2TT, UK*

Equation Labelling: Labels of the form (MAn) refer to equation n in the main article, while labels of the form (Sn) refer to equation n in this supplementary information.

S1 Dimensionless Equations

Substituting (MA40) into (MA10), the length of the filament can be determined via

$$\int_0^{L^*(t^*)/L} \cos\theta(s^*, t^*)\,\mathrm{d}s^* = \frac{H}{L} - \varepsilon^n R^*(\phi, L^*(t^*)/L, t^*), \tag{S1}$$

with $\varepsilon^n R^*(\phi, L^*(t^*)/L, t^*)$ the dimensionless filament radius on the build platform (at $s^* = L^*(t^*)/L$). While conservation of momentum equations (MA22) and conservation of mass (MA24) provide

$$\begin{aligned}
&\varepsilon^n \,\mathrm{Re}_{\mathrm{PL}} \Bigg(\varepsilon^n \frac{\partial}{\partial t^*}(u_s^* + v_s^*) + (\varepsilon^n\, u_r^* + v_r^*)\frac{\partial u_s^*}{\partial r^*} + \frac{(\varepsilon^n\, u_\phi^* + v_\phi^*)}{r^*}\frac{\partial u_s^*}{\partial \phi} \\
&\qquad + \frac{\varepsilon^n (u_s^* + v_s^*)}{L_s^*}\frac{\partial u_s^*}{\partial s^*} + \frac{\varepsilon^{2n}\kappa^*\,(u_s^* + v_s^*)}{L_s^*}\left(\sin\phi\, u_\phi^* - \cos\phi\, u_r^*\right)\Bigg) \\
&= \frac{\partial \sigma_{rs}^*}{\partial r^*} + \frac{1}{r^*}\frac{\partial \sigma_{\phi s}^*}{\partial \phi} + \frac{\varepsilon^{2n}}{L_s^*}\frac{\partial \sigma_{ss}^*}{\partial s^*} + \frac{\sigma_{rs}^*}{r^*} - \frac{2\varepsilon^n\,\kappa^*}{L_s^*}\left(\cos\phi\,\sigma_{rs}^* - \sin\phi\,\sigma_{\phi s}^*\right) \\
&\quad + \varepsilon^{2n}\frac{\mathrm{Re}_{\mathrm{PL}}}{\mathrm{Fr}^2}\cos\theta,
\end{aligned} \tag{S2a}$$

*Corresponding Author: a.tansell@bham.ac.uk

$$
\begin{aligned}
&\varepsilon^n \,\mathrm{Re}_{\mathrm{PL}} \left(\frac{\partial}{\partial t^*}(\varepsilon^n\, u_\phi^* + v_\phi^*) + (\varepsilon^n\, u_r^* + v_r^*)\frac{\partial u_\phi^*}{\partial r^*} + \frac{(\varepsilon^n\, u_\phi^* + v_\phi^*)}{r^*}\frac{\partial u_\phi^*}{\partial \phi} \right. \\
&\qquad \left. + \frac{\varepsilon^n (u_s^* + v_s^*)}{L_s^*}\frac{\partial u_\phi^*}{\partial s^*} + \frac{u_r^*(\varepsilon\, u_\phi^* + v_\phi^*)}{r^*} - \frac{\sin\phi\,(u_s^* + v_s^*)}{L_s^*}\left(\kappa^*\, u_s^* + \frac{\partial \theta}{\partial t^*}\right)\right) \\
&= \frac{\partial \sigma_{r\phi}^*}{\partial r^*} + \frac{1}{r^*}\frac{\partial \sigma_{\phi\phi}^*}{\partial \phi} + \frac{1}{L_s^*}\frac{\partial \sigma_{s\phi}^*}{\partial s^*} + \frac{2\,\sigma_{r\phi}^*}{r^*} + \frac{\varepsilon^n \kappa^*}{L_s^*}\left(\sin\phi\,(\sigma_{\phi\phi}^* - \sigma_{ss}^*) - \cos\phi\,\sigma_{r\phi}^*\right) \\
&\quad + \varepsilon^n\,\frac{\mathrm{Re}_{\mathrm{PL}}}{\mathrm{Fr}^2}\sin\theta\sin\phi,
\end{aligned}
\tag{S2b}
$$

$$
\begin{aligned}
&\varepsilon^n \,\mathrm{Re}_{\mathrm{PL}} \left(\frac{\partial}{\partial t^*}(\varepsilon^n\, u_r^* + v_r^*) + (\varepsilon^n\, u_r^* + v_r^*)\frac{\partial u_r^*}{\partial r^*} + \frac{(\varepsilon^n\, u_\phi^* + v_\phi^*)}{r^*}\frac{\partial u_r^*}{\partial \phi} \right. \\
&\qquad \left. + \frac{\varepsilon^n (u_s^* + v_s^*)}{L_s^*}\frac{\partial u_r^*}{\partial s^*} - \frac{u_\phi(\varepsilon^n\, u_\phi^* + v_\phi^*)}{r^*} + \frac{\cos\phi\,(u_s + v_s^*)}{L_s^*}\left(\kappa^*\, u_s^* + \frac{\partial \theta}{\partial t^*}\right)\right) \\
&= \frac{\partial \sigma_{rr}^*}{\partial r^*} + \frac{1}{r^*}\frac{\partial \sigma_{\phi r}^*}{\partial \phi} + \frac{1}{L_s^*}\frac{\partial \sigma_{sr}^*}{\partial s^*} + \frac{\sigma_{rr}^* - \sigma_{\phi\phi}^*}{r^*} + \frac{\varepsilon^n \kappa^*}{L_s^*}\left(\cos\phi\,(\sigma_{ss}^* - \sigma_{rr}^*) - \sin\phi\,\sigma_{\phi r}^*\right) \\
&\quad - \varepsilon^n\,\frac{\mathrm{Re}_{\mathrm{PL}}}{\mathrm{Fr}^2}\sin\theta\cos\phi,
\end{aligned}
\tag{S2c}
$$

$$
\frac{u_r^*}{r^*} + \frac{\partial u_r^*}{\partial r^*} + \frac{1}{r^*}\frac{\partial u_\phi^*}{\partial \phi} + \frac{1}{L_s^*}\frac{\partial u_s^*}{\partial s^*} - \frac{\varepsilon^n \kappa}{L_s^*}\left(\cos\phi\, u_r^* - \sin\phi\, u_\phi^*\right) = 0,
\tag{S2d}
$$

where (S2a), (S2b), and (S2c) are the $s-$, $\phi-$, and $r-$components of conservation of momentum, respectively, with

$$
\varepsilon^n = \frac{R_{\mathrm{N}}}{L}, \quad \mathrm{Re}_{\mathrm{PL}} = \frac{\rho\,(\bar{u}_{\mathrm{E}})^{2-n} L^n}{K}, \quad \text{and} \quad \mathrm{Fr} = \frac{\bar{u}_{\mathrm{E}}}{\sqrt{gL}},
\tag{S3}
$$

denoting the small aspect ratio, a generalised *Reynolds number* for a power-law fluid (a dimensionless number representing the ratio between inertial and viscous forces), and *Froude number* (a dimensionless number representing the ratio between inertial and gravitational forces), respectively, $L_s^* = 1 - \varepsilon^n\, r^* \kappa^* \cos\phi$, $\kappa^* = \partial\theta/\partial s^*$, and

$$
\sigma_{rr}^* = -p^* + 2\,\dot{\gamma}^{*^{n-1}}\frac{\partial u_r^*}{\partial r^*},
\tag{S4a}
$$

$$
\sigma_{\phi\phi}^* = -p^* + \frac{2}{r^*}\dot{\gamma}^{*^{n-1}}\left(\frac{\partial u_\phi^*}{\partial \phi} + u_r^*\right),
\tag{S4b}
$$

$$
\sigma_{ss}^* = -p^* + \frac{2}{L_s^*}\dot{\gamma}^{*^{n-1}}\left(\frac{\partial u_s^*}{\partial s^*} + \varepsilon^n \kappa^*\left(\sin\phi\, u_\phi^* - \cos\phi\, u_r^*\right)\right),
\tag{S4c}
$$

$$
\sigma_{\phi r}^* = \sigma_{r\phi}^* = \dot{\gamma}^{*^{n-1}}\left(\frac{\partial u_\phi^*}{\partial r^*} + \frac{1}{r^*}\left(\frac{\partial u_r^*}{\partial \phi} - u_\phi^*\right)\right),
\tag{S4d}
$$

$$
\sigma_{\phi s}^* = \sigma_{s\phi}^* = \dot{\gamma}^{*^{n-1}}\left(\frac{1}{r^*}\frac{\partial u_s}{\partial \phi} - \frac{\varepsilon^n}{L_s^*}\left(\sin\phi\left(\kappa^* u_s^* + \frac{\partial\theta}{\partial t^*}\right) - \varepsilon^n\,\frac{\partial u_\phi^*}{\partial s^*}\right)\right),
\tag{S4e}
$$

$$\sigma^*_{rs} = \sigma^*_{sr} = \dot{\gamma}^{*^{n-1}} \left(\frac{\partial u^*_s}{\partial r^*} + \frac{\varepsilon^n}{L^*_s} \left(\cos\phi \left(\kappa^* u^*_s + \frac{\partial \theta}{\partial t^*} \right) + \varepsilon^n \frac{\partial u^*_r}{\partial s^*} \right) \right), \tag{S4f}$$

where

$$\begin{aligned} \dot{\gamma}^{*^2} = &\frac{1}{2\varepsilon^{2n}} \left(\frac{1}{r^*} \frac{\partial u^*_s}{\partial \phi} + \frac{\varepsilon^n}{L^*_s} \left(\varepsilon^n \frac{\partial u^*_\phi}{\partial s^*} - \sin\phi \left(\kappa^* u^*_s + \frac{\partial \theta}{\partial t^*} \right) \right) \right)^2 \\ &+ \frac{1}{2\varepsilon^{2n}} \left(\frac{\partial u^*_s}{\partial r^*} + \frac{\varepsilon^n}{L^*_s} \left(\varepsilon^n \frac{\partial u^*_r}{\partial s^*} + \cos\phi \left(\kappa^* u^*_s + \frac{\partial \theta}{\partial t^*} \right) \right) \right)^2 \\ &+ 2 \left(\frac{\partial u^*_r}{\partial r^*} \right)^2 + \frac{2}{(r^*)^2} \left(\frac{\partial u^*_\phi}{\partial \phi} + u^*_r \right)^2 + \frac{1}{2} \left(\frac{\partial u^*_\phi}{\partial r^*} + \frac{1}{r^*} \left(\frac{\partial u^*_r}{\partial \phi} - u^*_\phi \right) \right)^2 \\ &+ \frac{2}{L^{*^2}_s} \left(\frac{\partial u^*_s}{\partial s^*} + \varepsilon^n \kappa^* \left(\sin\phi\, u^*_\phi - \cos\phi\, u^*_r \right) \right)^2 . \end{aligned} \tag{S5}$$

Following non-dimensionalisation, (MA7) and (MA18) retain their form. Substituting (MA40) into (MA29) we obtain the following dimensionless boundary condition

$$\sigma^*_{rr} n^*_r + \sigma^*_{r\phi} n^*_\phi + \sigma^*_{rs} n^*_s = 0, \qquad \text{on} \quad r^* = R^*(\phi, s^*, t^*), \tag{S6a}$$

$$\sigma^*_{\phi r} n^*_r + \sigma^*_{\phi\phi} n^*_\phi + \sigma^*_{\phi s} n^*_s = 0, \qquad \text{on} \quad r^* = R^*(\phi, s^*, t^*), \tag{S6b}$$

$$\sigma^*_{sr} n^*_r + \sigma^*_{s\phi} n^*_\phi + \varepsilon^{2n} \sigma^*_{ss} n^*_s = 0, \qquad \text{on} \quad r^* = R^*(\phi, s^*, t^*), \tag{S6c}$$

where

$$\begin{aligned} n^*_r &= (\Lambda^*)^{-\frac{1}{2}} R^* \tilde{L}^*_s, \\ n^*_\phi &= -(\Lambda^*)^{-\frac{1}{2}} \frac{\partial R^*}{\partial \phi} \tilde{L}^*_s, \\ n^*_s &= -(\Lambda^*)^{-\frac{1}{2}} R^* \frac{\partial R^*}{\partial s^*}, \end{aligned} \tag{S7}$$

are dimensionless components of the unit normal, with $\tilde{L}^*_s = 1 - \varepsilon^n R^* \kappa^* \cos\phi$, and

$$\Lambda^* = (R^*)^2 \left((\tilde{L}^*_s)^2 + \varepsilon^{2n} \left(\frac{\partial R^*}{\partial s^*} \right)^2 \right) + \left(\frac{\partial R^*}{\partial \phi} \right)^2 (\tilde{L}^*_s)^2. \tag{S8}$$

We also have kinematic boundary condition

$$u^*_r = \frac{\partial R^*}{\partial t^*} + \frac{u^*_\phi}{r^*} \frac{\partial R^*}{\partial \phi} + \frac{1}{L^*_s} \frac{\partial R^*}{\partial s^*} \left(u^*_s + \varepsilon^n R^* \frac{\partial \theta}{\partial t^*} \cos\phi \right), \qquad \text{at} \quad r^* = R^*(\phi, s^*, t^*), \tag{S9}$$

periodicity conditions within the cross-section

$$\begin{aligned} p^*(r^*, 0, s^*, t^*) &= p^*(r^*, 2\pi, s^*, t^*), \\ u^*_i(r^*, 0, s^*, t^*) &= u^*_i(r^*, 2\pi, s^*, t^*), \qquad \text{for} \quad i = r, \phi, s, \\ \frac{\partial u^*_i}{\partial j}(r^*, 0, s^*, t^*) &= \frac{\partial u^*_i}{\partial j}(r^*, 2\pi, s^*, t^*), \qquad \text{for} \quad i, j = r, \phi, s, \end{aligned} \tag{S10}$$

and

$$u_i^*(r^*,\phi^*,s^*,t^*) = 0, \qquad \text{for} \quad i = r,\phi, \quad \text{at} \quad r^* = 0, \tag{S11a}$$

$$R^*(\phi,s^*,t^*) = 1, \qquad \text{at} \quad s^* = 0, \tag{S11b}$$

$$u_s^*(r^*,\phi,s^*,t^*) = \frac{u_{\mathrm{E}}}{\bar{u}_{\mathrm{E}}}, \qquad \text{at} \quad s^* = 0, \tag{S11c}$$

$$u_\phi^*(r^*,\phi,s^*,t^*) = 0, \qquad \text{at} \quad s^* = 0, \tag{S11d}$$

$$u_s^*(r^*,\phi,s^*,t^*) = \frac{U_{\mathrm{N}}}{\bar{u}_{\mathrm{E}}}, \qquad \text{at} \quad s^* = \frac{L^*(t^*)}{L}, \tag{S11e}$$

$$\theta(s^*,t^*) = \frac{\pi}{2}, \qquad \text{at} \quad s^* = \frac{L^*(t^*)}{L}. \tag{S11f}$$

S2 Slender-Body Asymptotics

Under the assumption that all leading-order variables are independent of ϕ, the corresponding components of the stress tensor and the unit normal used in the main article are given by

$$\sigma_{rr}^{(0)} = -p^{(0)} + 2\left(\dot{\gamma}^{n-1}\right)^{(0)} \frac{\partial u_r^{(0)}}{\partial r}, \tag{S12a}$$

$$\sigma_{rr}^{(1)} = -p^{(1)} + 2\left(\dot{\gamma}^{n-1}\right)^{(0)} \frac{\partial u_r^{(1)}}{\partial r} + 2\left(\dot{\gamma}^{n-1}\right)^{(1)} \frac{\partial u_r^{(0)}}{\partial r}, \tag{S12b}$$

$$\sigma_{rr}^{(2)} = -p^{(2)} + 2\left(\dot{\gamma}^{n-1}\right)^{(0)} \frac{\partial u_r^{(2)}}{\partial r} + 2\left(\dot{\gamma}^{n-1}\right)^{(1)} \frac{\partial u_r^{(1)}}{\partial r} + 2\left(\dot{\gamma}^{n-1}\right)^{(2)} \frac{\partial u_r^{(0)}}{\partial r}, \tag{S12c}$$

$$\sigma_{\phi\phi}^{(0)} = -p^{(0)} + \frac{2}{r}\left(\dot{\gamma}^{n-1}\right)^{(0)} u_r^{(0)}, \tag{S12d}$$

$$\sigma_{\phi\phi}^{(1)} = -p^{(1)} + \frac{2}{r}\left(\dot{\gamma}^{n-1}\right)^{(0)} \left(\frac{\partial u_\phi^{(1)}}{\partial \phi} + u_r^{(1)}\right) + \frac{2}{r}\left(\dot{\gamma}^{n-1}\right)^{(1)} \left(\frac{\partial u_\phi^{(0)}}{\partial \phi} + u_r^{(0)}\right), \tag{S12e}$$

$$\sigma_{ss}^{(0)} = -p^{(0)} + 2\left(\dot{\gamma}^{n-1}\right)^{(0)} \frac{\partial u_s^{(0)}}{\partial s}, \tag{S12f}$$

$$\begin{aligned}\sigma_{ss}^{(1)} = -p^{(1)} &+ 2\left(\dot{\gamma}^{n-1}\right)^{(0)} \left(\frac{\partial u_s^{(1)}}{\partial s} + \kappa^{(0)}\left(\sin\phi\, u_\phi^{(0)} - \cos\phi\left(u_r^{(0)} - r\frac{\partial u_s^{(0)}}{\partial s}\right)\right)\right) \\ &+ 2\left(\dot{\gamma}^{n-1}\right)^{(1)} \frac{\partial u_s^{(0)}}{\partial s},\end{aligned} \tag{S12g}$$

$$\sigma_{\phi r}^{(0)} = \sigma_{r\phi}^{(0)} = \left(\dot{\gamma}^{n-1}\right)^{(0)} \left(\frac{\partial u_\phi^{(0)}}{\partial r} - \frac{u_\phi^{(0)}}{r}\right), \tag{S12h}$$

$$\begin{aligned}\sigma_{\phi r}^{(1)} = \sigma_{r\phi}^{(1)} = \left(\dot{\gamma}^{n-1}\right)^{(0)} \left(\frac{\partial u_\phi^{(1)}}{\partial r} + \frac{1}{r}\left(\frac{\partial u_r^{(1)}}{\partial \phi} - u_\phi^{(1)}\right)\right) \\ + \left(\dot{\gamma}^{n-1}\right)^{(1)} \left(\frac{\partial u_\phi^{(0)}}{\partial r} - \frac{u_\phi^{(0)}}{r}\right),\end{aligned} \tag{S12i}$$

$$\begin{aligned}\sigma_{\phi r}^{(2)} = \left(\dot{\gamma}^{n-1}\right)^{(2)} \left(\frac{\partial u_\phi^{(0)}}{\partial r} - \frac{u_\phi^{(0)}}{r}\right) + \left(\dot{\gamma}^{n-1}\right)^{(1)} \left(\frac{\partial u_\phi^{(1)}}{\partial r} + \frac{1}{r}\left(\frac{\partial u_r^{(1)}}{\partial \phi} - u_\phi^{(1)}\right)\right) \\ + \left(\dot{\gamma}^{n-1}\right)^{(0)} \left(\frac{\partial u_\phi^{(2)}}{\partial r} + \frac{1}{r}\left(\frac{\partial u_r^{(2)}}{\partial \phi} - u_\phi^{(2)}\right)\right),\end{aligned} \tag{S12j}$$

$$\sigma_{\phi s}^{(0)} = \sigma_{s\phi}^{(0)} = 0, \tag{S12k}$$

$$\sigma_{\phi s}^{(1)} = \sigma_{s\phi}^{(1)} = \left(\dot{\gamma}^{n-1}\right)^{(0)} \left(\frac{1}{r}\frac{\partial u_s^{(1)}}{\partial \phi} - \sin\phi\left(\kappa^{(0)} u_s^{(0)} + \frac{\partial \theta^{(0)}}{\partial t}\right)\right), \tag{S12l}$$

$$\begin{aligned}\sigma_{\phi s}^{(2)} = \left(\dot{\gamma}^{n-1}\right)^{(0)} \left(\frac{1}{r}\frac{\partial u_s^{(2)}}{\partial \phi} + \frac{\partial u_\phi^{(0)}}{\partial s} - r\kappa^{(0)}\cos\phi\sin\phi\left(\kappa^{(0)} u_s^{(0)} + \frac{\partial \theta^{(0)}}{\partial t}\right)\right. \\ \left. - \sin\phi\left(\kappa^{(1)} u_s^{(0)} + \kappa^{(0)} u_s^{(1)} + \frac{\partial \theta^{(1)}}{\partial t}\right)\right) + \left(\dot{\gamma}^{n-1}\right)^{(1)} \left(\frac{1}{r}\frac{\partial u_s^{(1)}}{\partial \phi}\right. \\ \left. - \sin\phi\left(\kappa^{(0)} u_s^{(0)} + \frac{\partial \theta^{(0)}}{\partial t}\right)\right),\end{aligned} \tag{S12m}$$

$$\sigma_{rs}^{(0)} = \sigma_{sr}^{(0)} = \left(\dot{\gamma}^{n-1}\right)^{(0)} \frac{\partial u_s^{(0)}}{\partial r}, \tag{S12n}$$

$$\sigma_{rs}^{(1)} = \sigma_{sr}^{(1)} = \left(\dot{\gamma}^{n-1}\right)^{(0)} \left(\frac{\partial u_s^{(1)}}{\partial r} + \cos\phi\left(\kappa^{(0)} u_s^{(0)} + \frac{\partial \theta^{(0)}}{\partial t}\right)\right) + \left(\dot{\gamma}^{n-1}\right)^{(1)} \frac{\partial u_s^{(0)}}{\partial r}, \tag{S12o}$$

$$\begin{aligned}\sigma_{rs}^{(2)} = \sigma_{sr}^{(2)} = \left(\dot{\gamma}^{n-1}\right)^{(0)} \left(\frac{\partial u_s^{(2)}}{\partial r} + \frac{\partial u_r^{(0)}}{\partial s} + r\kappa^{(0)}\cos^2\phi\left(\kappa^{(0)} u_s^{(0)} + \frac{\partial \theta^{(0)}}{\partial t}\right)\right. \\ \left. + \cos\phi\left(\kappa^{(1)} u_s^{(0)} + \kappa^{(0)} u_s^{(1)} + \frac{\partial \theta^{(1)}}{\partial t}\right)\right) + \left(\dot{\gamma}^{n-1}\right)^{(1)} \left(\frac{\partial u_s^{(1)}}{\partial r}\right. \\ \left. + \cos\phi\left(\kappa^{(0)} u_s^{(0)} + \frac{\partial \theta^{(0)}}{\partial t}\right)\right) + \left(\dot{\gamma}^{n-1}\right)^{(2)} \frac{\partial u_s^{(0)}}{\partial r},\end{aligned} \tag{S12p}$$

$$\tilde{n}_r^{(0)} = R^{(0)}, \tag{S12q}$$

$$\tilde{n}_r^{(1)} = R^{(1)} - \left(R^{(0)}\right)^2 \kappa^{(0)} \cos\phi, \tag{S12r}$$

$$\tilde{n}_r^{(2)} = R^{(2)} - R^{(0)} \cos\phi \left(2R^{(1)}\kappa^{(0)} + R^{(0)}\kappa^{(1)}\right), \tag{S12s}$$

$$\tilde{n}_\phi^{(0)} = 0, \tag{S12t}$$

$$\tilde{n}_\phi^{(1)} = -\frac{\partial R^{(1)}}{\partial \phi}, \tag{S12u}$$

$$\tilde{n}_\phi^{(2)} = -\frac{\partial R^{(2)}}{\partial \phi} + R^{(0)}\kappa^{(0)} \cos\phi \frac{\partial R^{(1)}}{\partial \phi}, \tag{S12v}$$

$$\tilde{n}_s^{(0)} = -R^{(0)} \frac{\partial R^{(0)}}{\partial s}, \tag{S12w}$$

$$\tilde{n}_s^{(1)} = -R^{(1)} \frac{\partial R^{(0)}}{\partial s} - R^{(0)} \frac{\partial R^{(1)}}{\partial s}, \tag{S12x}$$

$$\tilde{n}_s^{(2)} = -R^{(2)} \frac{\partial R^{(0)}}{\partial s} - R^{(1)} \frac{\partial R^{(1)}}{\partial s} - R^{(0)} \frac{\partial R^{(2)}}{\partial s}. \tag{S12y}$$

S3 Curve Fittings

Table S1 presents quadratic polynomials fitted to the experimental filament radius, R_L, and filament length, L, as the air pressure, ΔP, applied to Nivea Crème was varied (for preliminary model validation purposes in the main article).

**Functional Form**	**a**	**b**	**c**
$R_L = a\,(\Delta P)^2 + b\,\Delta P + c$	-1.35×10^{-12}	1.65×10^{-7}	4.71×10^{-3}
$L = a\,(\Delta P)^2 + b\,\Delta P + c$	-1.08×10^{-9}	1.18×10^{-4}	-3.21

Table S1: Constant coefficients of curve-fitting for experimental filament radius, R_L (row 1), and filament length, L (row 2), as air pressure, ΔP, applied to Nivea Crème is varied, obtained via regression analysis conducted in MATLAB® and captured in Figures 6(a) and 6(b), respectively, in the main article.
